# Cardiovascular disease risk communication and prevention: a meta-analysis

**DOI:** 10.1093/eurheartj/ehae002

**Published:** 2024-01-19

**Authors:** Mina Bakhit, Samantha Fien, Eman Abukmail, Mark Jones, Justin Clark, Anna Mae Scott, Paul Glasziou, Magnolia Cardona

**Affiliations:** Institute for Evidence-Based Healthcare, Faculty of Health Sciences and Medicine, Bond University, Robina, QLD, Australia; School of Health, Medical and Applied Sciences, Central Queensland University, Mackay, QLD, Australia; Institute for Evidence-Based Healthcare, Faculty of Health Sciences and Medicine, Bond University, Robina, QLD, Australia; Institute for Evidence-Based Healthcare, Faculty of Health Sciences and Medicine, Bond University, Robina, QLD, Australia; Institute for Evidence-Based Healthcare, Faculty of Health Sciences and Medicine, Bond University, Robina, QLD, Australia; Institute for Evidence-Based Healthcare, Faculty of Health Sciences and Medicine, Bond University, Robina, QLD, Australia; Institute for Evidence-Based Healthcare, Faculty of Health Sciences and Medicine, Bond University, Robina, QLD, Australia; Institute for Evidence-Based Healthcare, Faculty of Health Sciences and Medicine, Bond University, Robina, QLD, Australia

**Keywords:** Cardiovascular risk, Risk scoring, Risk communication, Absolute risk, Systematic review

## Abstract

**Background and Aims:**

Knowledge of quantifiable cardiovascular disease (CVD) risk may improve health outcomes and trigger behavioural change in patients or clinicians. This review aimed to investigate the impact of CVD risk communication on patient-perceived CVD risk and changes in CVD risk factors.

**Methods:**

PubMed, Embase, and PsycINFO databases were searched from inception to 6 June 2023, supplemented by citation analysis. Randomized trials that compared any CVD risk communication strategy versus usual care were included. Paired reviewers independently screened the identified records and extracted the data; disagreements were resolved by a third author. The primary outcome was the accuracy of risk perception. Secondary outcomes were clinician-reported changes in CVD risk, psychological responses, intention to modify lifestyle, and self-reported changes in risk factors and clinician prescribing of preventive medicines.

**Results:**

Sixty-two trials were included. Accuracy of risk perception was higher among intervention participants (odds ratio = 2.31, 95% confidence interval = 1.63 to 3.27). A statistically significant improvement in overall CVD risk scores was found at 6–12 months (mean difference = −0.27, 95% confidence interval = −0.45 to −0.09). For primary prevention, risk communication significantly increased self-reported dietary modification (odds ratio = 1.50, 95% confidence interval = 1.21 to 1.86) with no increase in intention or actual changes in smoking cessation or physical activity. A significant impact on patients’ intention to start preventive medication was found for primary and secondary prevention, with changes at follow-up for the primary prevention group.

**Conclusions:**

In this systematic review and meta-analysis, communicating CVD risk information, regardless of the method, reduced the overall risk factors and enhanced patients’ self-perceived risk. Communication of CVD risk to patients should be considered in routine consultations.

## Structured Graphical Abstract

**Structured Graphical Abstract:**
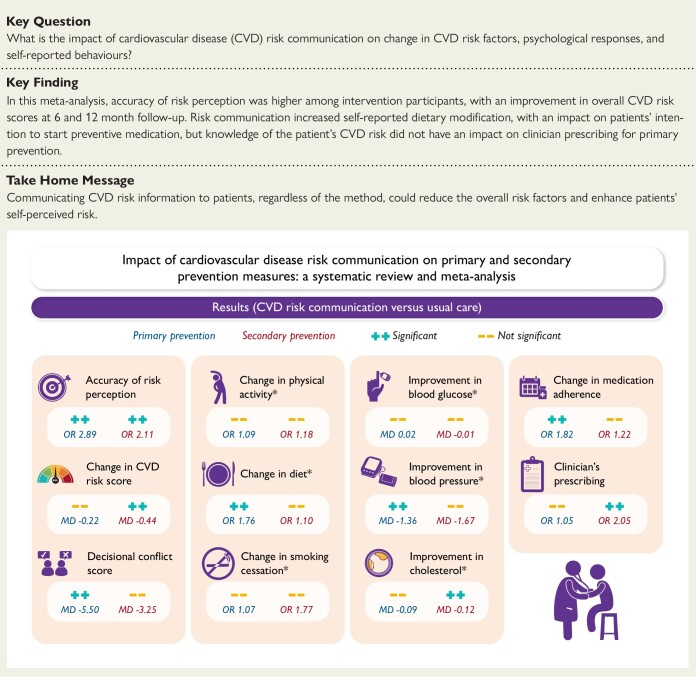
The impact of cardiovascular disease risk communication on primary and secondary prevention measures compared to usual care. CVD, cardiovascular disease; MD, mean difference; OR, odds ratio. *Actual changes.

**See the editorial comment for this article ‘The art of deciphering and communicating cardiovascular risk: getting it right', by S.U. Khan *et al*., https://doi.org/10.1093/eurheartj/ehae103.**

## Introduction

The increasing prevalence of cardiovascular disease (CVD) and associated morbidity is now considered a global emergency. Cardiovascular disease mortality is estimated to account for a third of all deaths worldwide (17.9 million per year according to the World Health Organization).^1^ The corresponding Australian estimate is 25%,^2^ with heart attacks and strokes responsible for 85% of the yearly deaths.^2^ Sedentary lifestyles, smoking, unhealthy diet, and poor screening behaviour are largely responsible for the escalation of this problem.^2^

For decades, clinicians and researchers have implemented effective measures to reduce, diagnose, and treat CVD.^3–5^ This includes implementing public education campaigns aimed at improving awareness of the potential preventability of CVDs and accessibility to management.^3–5^ Cardiovascular disease risk calculators to estimate individualized risk have also played a role in either preventing or modifying risk factors.^6^ Different international guidelines usually recommend to communicate individual CVD risk, which is calculated by combining risk factors in an empirical equation, as a first step to establish behaviour modification to decrease CVD risk even for asymptomatic people. Risk communication is challenging as multiple factors appear to affect risk perception, for example, how best to visually present this information and which numerical format to use. Timeframe (lifetime, 10 year) of the risk can also influence the perception of risk severity and intention to initiate treatment even in educated populations.^6,7^

Various medical specialties have examined the gap between communicating risk information and inducing behaviour change. However, the effectiveness of different strategies to reduce this gap has shown mixed results.^8,9^

Several systematic reviews have investigated the effect of risk information on clinical outcomes either by looking at the effect of personalized risk information as general descriptors^10^ or providing CVD risk scores (irrespective of the model used e.g. Framingham) or comparing the effect of the different calculators on accuracy of risk perception.^11,12^ The systematic reviews reported mixed results and the impact of risk communication interventions on changes in the accuracy of risk perception, patient intentions and actual behaviour change, and clinician’s medications prescribing (in response to the knowledge of their patients’ CVD risk) remains unclear. Hence, an updated synthesis with the inclusion of results from newly published articles was warranted to enhance understanding of the total effects of risk communication on patients and doctors for primary and secondary prevention.

This review investigated the following research questions: (i) What is the impact of CVD risk communication on patient-perceived CVD risk, actual change in CVD risk factors, psychological responses, and self-reported behaviours? (ii) What is the impact of CVD risk knowledge on clinician’s prescribing behaviour?

## Methods

This systematic review is reported following the Preferred Reporting Items for Systematic Reviews and Meta-Analyses (PRISMA) statement.^13^ The protocol was developed prospectively and registered on the Open Science Framework (https://doi.org/10.17605/OSF.IO/SNAKV).

### Eligibility criteria

#### Participants

We included studies of adults aged 30 years and above with (secondary prevention) or without (primary prevention) established CVD. Studies of adults with genetic predisposition of CVD or with a sample size less than 40 were excluded for the validity of normal approximation of estimates.^14^

### Interventions and comparators

We included trials that compared any type of CVD risk communication strategy covering online, paper-based, or verbal administration of any scoring system or tool presenting global CVD risk score or the risk of any specific clinical CVD events (e.g. heart attack, stroke, or atrial fibrillation). This was then compared with usual care with or without any CVD risk communication strategy including comparisons against different risk communication formats (e.g. visual, verbal, and numeric) or communication strategies (e.g. threat and efficacy).

### Setting

Eligible interventions were those implemented in any health setting and delivered by any healthcare provider (e.g. cardiologist, general practitioner, nurse, or pharmacist).

### Types of outcome measures

#### Primary outcome

- Accuracy of risk perception (patient-reported) as defined and reported eligible by study authors, which could be reported using a scale, numerical, or categorial and then assessed for its accuracy (where the numerator is the self-perception of risk and the denominator is the objectively calculated risk, or where categories of low–medium–high are assigned to % risk levels).

#### Secondary outcomes

##### Participant-reported outcomes

- Psychological responses to CVD risk information (e.g. decisional conflict and depression).
- Behaviour changes either intention to change (e.g. smoking cessation, exercise, and start medications) or actual reported and recorded changes (e.g. quit smoking, lost weight, and dietary changes).

##### Clinician-reported outcomes

- Change in predicted global CVD risk or event rates.
- Changes in blood pressure, lipids, and glucose levels.
- Prescribing of new/additional medications (e.g. lipid-lowering and/or antihypertensive medications) or lifestyle changes (e.g. smoking cessation).

### Study design

We included randomized controlled trials (RCTs) of any design (e.g. parallel, cluster, and crossover) if at least one of the primary or secondary outcomes of interest was reported. Excluded were as follows: observational studies, pre–post or qualitative designs, or studies using hypothetical case scenarios of risk perception. Reviews of primary studies (e.g. systematic reviews and literature reviews) were excluded, but the reference lists of any relevant reviews were checked for any additional, relevant primary studies.

### Timeframe

We included eligible outcomes reported immediately after the intervention and other follow-up timeframes ranging from 2 weeks to over 12 months.

### Search strategies

#### Database search

PubMed (MEDLINE), Embase (Elsevier), and PsycINFO (OVID) were searched from inception to 6 June 2023. One of the review authors (J.C.), designed the search string in PubMed, refined and pilot tested with two other authors (M.B. and M.C.), and then translated it for use in the other databases using the Polyglot Search Translator.^15^ The complete search strategy for all databases is provided in Supplementary data online, *Box S1*. No restrictions by language or publication date were imposed, but only publications that were available in full were includable. Conference abstracts were excluded unless they had a clinical trial registry record, or other public report, with the additional information required for inclusion. We supplemented our search with forward and backward citation searches of included studies and by contacting authors of studies not yet published, which we identified in conference abstracts or randomized trial protocols.

#### Study selection and screening

**Figure 1:**
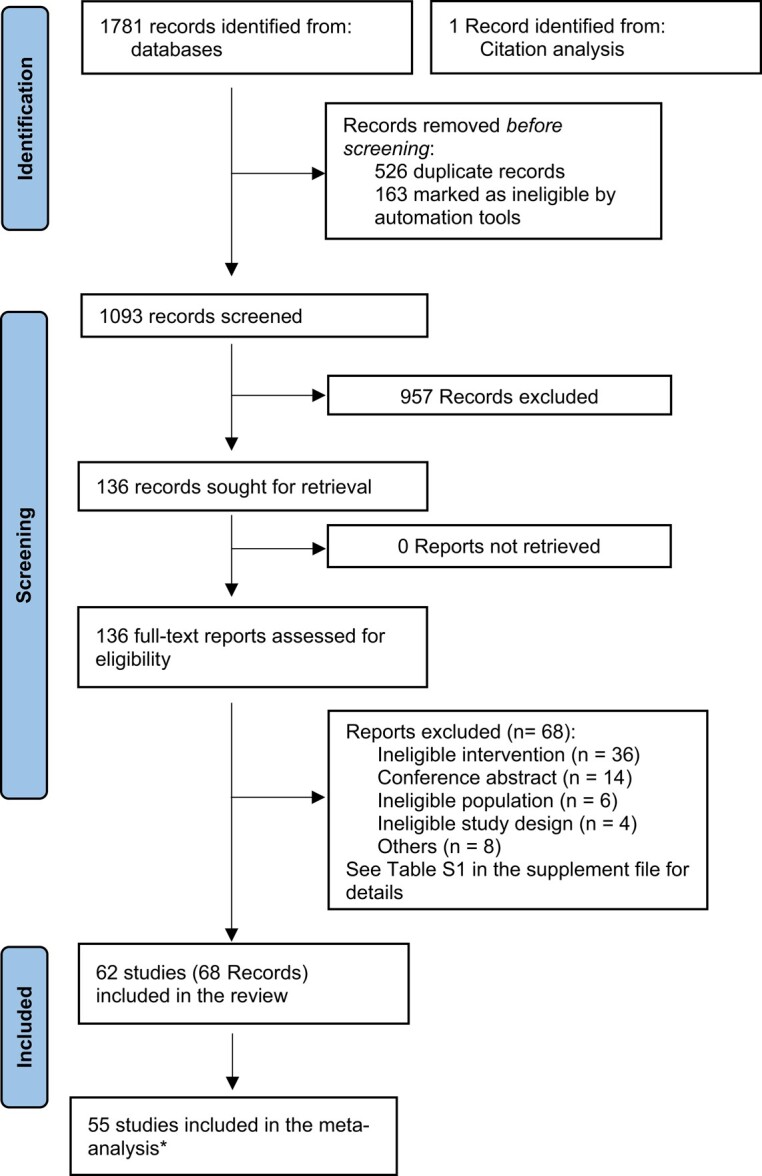
Preferred Reporting Items for Systematic Reviews and Meta-Analyses flow diagram. *At least one outcome in the article was included in the meta-analysis. Adapted from: Page MJ, McKenzie JE, Bossuyt PM, Boutron I, Hoffmann TC, Mulrow CD, *et al*. The PRISMA 2020 statement: an updated guideline for reporting systematic reviews. BMJ 2021; 372:n71. doi: 10.1136/bmj.n71

Two pairs of review authors (M.B. and E.A. or M.C. and S.F.) independently screened the title and abstract of retrieved records against the inclusion criteria. Screening was conducted using the *Screenatron* feature of the Systematic Review Accelerator.^16^ Disputes were identified using the *Disputatron* feature of the Systematic Review Accelerator and were resolved by discussion or by consulting a third author (M.C. or P.G.).^15,16^ See *Figure 1* for the PRISMA flow diagram outlining the selection process and Supplementary data online, *Table S1* for the complete list of excluded full-text articles with reasons for exclusions.

### Data extraction

A data extraction form was piloted on two of the included studies and modified based on feedback from within the team as required. Two pairs of review authors (M.B. and E.A. or M.C. and S.F.) independently extracted the following data:

- Study characteristics: author, publication year, country, setting, and design.
- Participants’ characteristics: target group setting, risk factors, sample size, age, sex, and cardiovascular risk score.
- Intervention and comparator description: type of risk communication strategy, who delivered the risk information, how, where, and when the information was delivered (as per the TIDieR framework^17^).
- Primary and secondary outcomes: as reported above.

### Assessment of risk of bias in included studies

Paired authors (M.B. and E.A. or M.C. and S.F.) independently assessed the risk of bias for each included RCT using the Cochrane Risk of Bias 1.0 tool.^18^ Risk of Bias Tool 1.0 was used in preference to the Risk of Bias Tool 2.0 as the former allows the assessment of biases from conflict of interest and funding (under the domain: other sources of bias), whilst the latter does not. The following domains were assessed: random sequence generation, allocation concealment, blinding (participants and personnel), blinding (outcome assessment), incomplete outcome data, selective reporting, and other sources of bias (e.g. funding and reported conflicts of interests). Each potential source of bias was graded as low, high, or unclear, and each judgement was supported by a quote from the relevant study. Any disagreements were resolved by discussion between screeners or by referring to a senior author (M.C. or P.G.).

### Measurement of effect and data synthesis

Where feasible, dichotomous data were expressed as odds ratios (ORs) or risk ratios with 95% confidence intervals (CIs). Continuous data were expressed as mean differences (MDs) or standardized MDs with 95% CIs. Meta-analysis was only undertaken when meaningful (i.e. when ≥2 studies or comparisons reported the same outcome) using the individual as the unit of analysis, where possible, and due to the anticipated heterogeneity among included studies, a random effects model was used (DerSimonian and Laird random effects method). We used the *I*^2^ statistic to measure heterogeneity among the included trials. Publication bias was intended to be assessed using a funnel plot, provided there were greater than 10 trials included in the analysis. When meta-analysis was not feasible, studies were plotted to facilitate interpretation and narrative explanation was provided.^19^

For psychological response data, the only meta-analysable outcome under this domain was decisional conflict, a concept encompassing ambiguity on the next steps to take on lifestyle modifications. Decisional conflict scales generally covered subcomponents of uncertainty, lack of clarity, information deficits, perception of support levels, and quality of the decision. The outcome was measured as a difference between follow-up and baseline score points.

We also intended to analyse the effects by the following *a priori*-defined subgroups, when feasible: type of comparator (e.g. usual care or another risk communication strategy), setting, and time to follow-up. We intended to analyse the data according to the TiDIER intervention components and to conduct a sensitivity analysis by including vs. excluding studies with three or more Cochrane risk of bias domains rated at high risk of bias; however, it was not possible due to the paucity of data. We reported the intervention components in Supplementary data online, *Table S2*.

## Results

### Included studies

Our search identified 1782 records, of which 1093 remained after duplicates were removed. After full-text screening of 136 records, 62 studies reported in 68 articles met our inclusion criteria.^20–86^ A total of 55 studies were included in the meta-analysis (reporting at least one outcome), and seven studies did not contribute to the meta-analysis^22,31,32,41,49,69,73^ (*Figure 1*).

### Characteristics of included studies

Most studies were individually randomized trials of face-to-face single-component interventions conducted in primary care in Europe and the USA (*Table 1* and Supplementary data online, *Table S3*).

**Table 1:** Summary characteristics of included studies

	Number of studies (references)
Study design	
Randomized controlled trials (RCTs)	44^21–23,25–27,29–33,35,37–42,47–52,55,58–60,64,66,67, 70–72,74,75,80–86^
Cluster RCTs	18^20,24,28,34,43,45,46,54,56,57,62,63,65,73,76–79^
Study location	
Europe^a^	26^20,21,23,24,28–31,38,42,43,45,46,54–58,66,70–74,76, 78,86^
North America	22^22,32,33,37,39–41,47,50–52,59,60,64,65,69,75,79–82^
Oceania	9^25,26,35,49,62,67,77,83,84^
South America	4^27,34,48,63^
Africa	1^85^
Number of intervention arms	
Two arm trials	47^20,21,24,26–30,32–35,37–41,43,45–47,50–52,57–60, 62–67,69,71,73,75–82,85,86^
Three or more arms	15^22,23,25,31,42,48,49,54–56,70,72,74,83,84^
Type of cardiovascular prevention	
Primary prevention	42^20–25,27–33,38,40,41,43,45,46,49,50,55,57,60,63–65, 69–75,77–79,82–86^
Secondary prevention	16^26,34,35,37,39,42,47,48,51,52,54,58,59,66,67,81^
Mixed	4^56,62,76,80^
Setting	
Primary care	33^20,21,24,27,31,34,37,42,43,45,46,50,51,54–57,60, 62–67,70,73–78,81,86^
Community	14^22,23,25,33,38,48,49,52,71,72,80,83–85^
Secondary care	7^29,30,32,58,69,79,82^
Tertiary care	5^26,35,39–41^
Mixed settings	3^28,47,59^
Intervention description	
Mode of delivery	
Face-to-face	41^20–22,24,26–35,37–41,43,45–52,54–58,62,63,66,73–76,78^
Remote^b^	15^23,25,42,59,64,65,67,69–72,82–85^
Mixed	6^60,77,79–81,86^
Type of intervention provider^c^	
General practitioners^d^	15^21,29–31,35,42,43,46,54,57,59,62,76,78,86^
Specialists	13^24,28,37,39–41,47,49–51,63,75,79^
Researchers	15^23,25,48,55,65–67,69–72,82–85^
Allied health	11^20,22,26,27,32,45,52,58,73,74,81^
Mixed	4^34,56,77,80^
Other	3^33,60,64^
Target participants	
Patients	32^22,23,25,31–33,37,38,42,49,52,55,58–60,64–67, 69–72,74,77,80–86^
Clinicians	13^28,41,43,46–48,50,51,54,56,57,75,76^
Mixed^e^	17^20,21,24,26,27,29,30,34,35,39,40,45,62,63,73,78,79^
Structure of interventions	
Single component	40^21–23,25–27,31,32,37–41,43,46–48,50–52,54–56,58, 63,65,66,69–76,79,82–84,86^
Multi-faceted	22^20,24,28–30,33–35,42,45,49,57,59,60,62,64, 67,77,78,80,81,85^

^a^Including one study with multi-EU countries.

^b^Including online/phone/mail.

^c^One study did not report the type of provider.

^d^Including family doctors.

^e^Two studies targeted system/organisational changes.

### Intervention descriptions

Of note, 22 interventions in this review were multi-component (*Table 1* and Supplementary data online, *Table S2*) and elements went beyond the communication of the risk, being supplemented with other strategies such as educational materials for patients to keep, motivational counselling, attendance to physical activity sessions, knowledge re-testing, interdisciplinary referrals, prescriptions, and follow-up appointments to check on progress. Fifteen trials used some form of decision aid or shared decision-making approach.^21,29,30,39,40,43,45–47,51,63,69,79,82,85^

### Risk of bias

**Figure 2:**
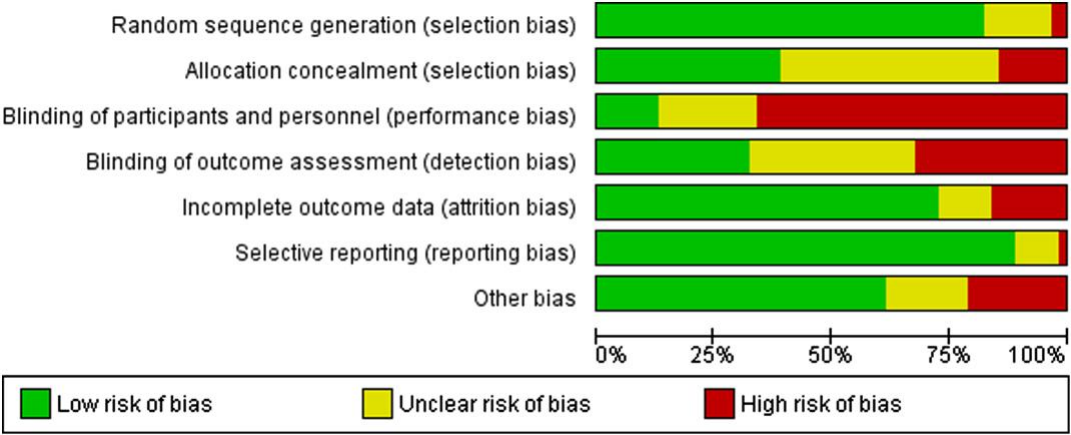
‘Risk of bias graph’ illustrates authors’ judgements about each risk of bias item presented as percentages across all included studies

The studies generally had low risk of selection bias with appropriate random sampling, low attrition rates, and complete reporting of intended outcomes. Some concerns were apparent on allocation concealment in almost half of the studies. High risk of bias was found in 41 studies that did not blind participants and/or personnel, with some concerns or unclear reporting for the remaining studies. Risk of bias for blinding of outcome assessment was rated as high or unclear for 20 and 22 studies, respectively. A total of 13 articles did not report on conflicts of interest or funding sources or both (*Figure 2* and Supplementary data online, *Figure S1*).

We were able to produce funnel plots to assess the potential for reporting bias for five outcomes, which are shown in Supplementary data online, *Box S2* (accuracy of risk perception, changes in CVD risk score, actual changes in medication, blood pressure, and cholesterol changes). Visual inspection of the plots indicated the potential for publication bias related to studies reporting these outcomes.

### Primary outcomes

#### Accuracy of risk perception

##### Meta-analysable studies

**Figure 3:**
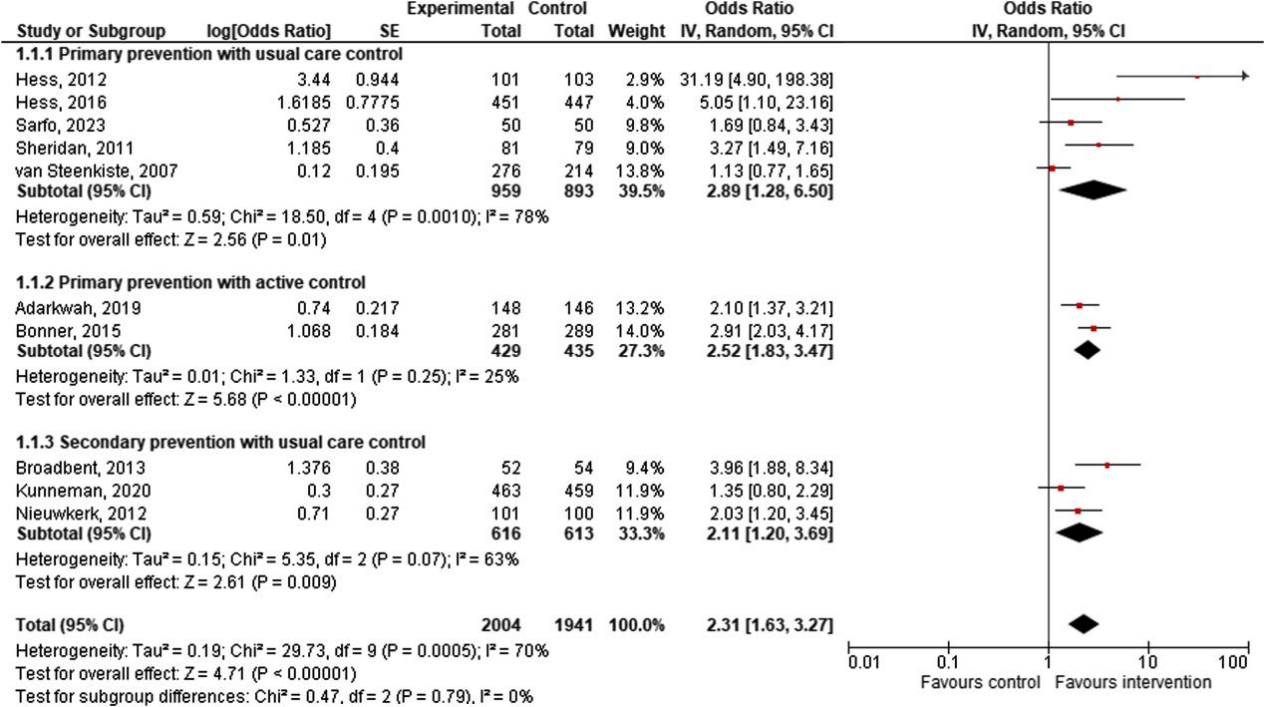
Accuracy of risk perception (*n* = 10 studies). CI, confidence interval; SE, standard error

The accuracy of risk perception assessed immediately after the risk communication in 10/55 studies was higher among intervention participants with or without established disease (10 studies, pooled estimate OR = 2.31, 95% CI = 1.63, 3.27) than among the control participants, although heterogeneity was high (*I*^2^ = 70%). This was apparent regardless of whether the control strategy was usual care or active control (*Figure 3*). A test for subgroup difference showed no evidence that the effect of intervention differed across primary versus secondary prevention subgroups (*P* = .79). The high heterogeneity for primary prevention vs. usual care was related to settings, personnel administering the intervention, and intervention components.

##### Non-meta-analysable studies

Among the eight non-meta-analysable studies for this outcome (*Table 2* and Supplementary data online, *Table S4*), only three primary prevention interventions led to significantly more accurate risk perception: two trials using decision aids with outcomes at 3- and 6-month follow-up, respectively^30,63^ and a multi-component study where the Heart Age message achieved higher accuracy at 4 weeks than intervention without the CVD risk communication.^72^

**Table 2:** A summary of findings for results of non-meta-analysable studies for primary and secondary outcomes

	Immediate	2 w	1 M	2–3 M	6–9 M	12 M
**Primary outcomes**						
Accurate risk perception (*n* = 8)	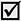 ? 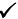 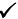 ^30,51,71,72^		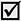 ^72^	?? 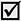 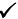 ^22,51,63,70^	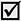 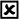 ? ^29,30,51^	
**Secondary outcomes**						
Change in CVD risk (*n* = 10)			? ^32^	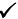 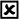 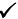 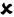 ^43,66,74,81^	? ^49^	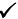 ? 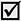 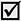 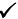 ^20,49,50,52,56^
Psychological response (*n* = 9)	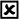 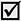 ^25,71^	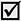 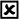 ^72,78^	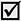 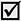 ^25,42^	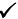 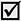 ^66,70^	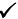 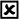 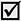 ^42,58,78^	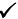 ^31^
**Smoking**						
Intention to change (*n* = 3)	? 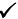 ^30,69^	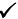 ^25^			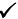 ^30^	
Actual change (*n* = 15)				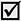 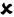 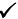 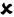 ^43,70,74,81^	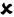 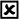 ? ^58,59,78^	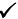 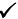 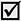 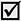 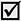 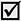 ? 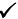 ^20,27,34,43,45,48,49,52,67^
**Physical activity**						
Intention to change (*n* = 2)		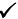 ^25^			? ^69^	
Actual change (*n* = 3)			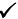 ^32^		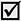 ^60^	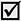 ^48^
**Dietary**						
Intention to change (*n* = 3)	? ^69^	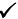 ^25^	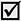 ^72^			
Actual change (*n* = 3)			? 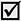 ^32,72^	? ^32^	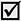 ^59^	
**Medication adherence**						
Intention to change (*n* = 1)					? ^69^	
Actual change (*n* = 1)						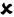 ^76^
**CVD risk factors**						
Blood pressure (*n* = 5)				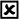 ^54^	? 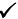 ^49,57^	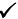 ? 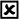 ^20,49,52^
Lipids (*n* = 4)			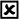 ^73^		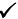 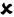 ^49,65^	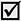 ^c^ ^52^
Glucose^b^ (*n* = 0)						
Clinicians’ prescribing (*n* = 3)					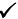 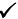 ^a^ ^57,59^	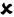 ^28^

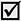
 Favours risk communication *P* < .05.

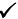
 Favours risk communication non-significant.

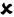
 Against risk communication non-significant.

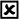
 Against risk communication *P* < .05.

? Not clearly reported.

^a^Significant for antihypertensive medications and non-significant for lipid-lowering medications.

^b^All studies were meta-analysed.

^c^Only for HDL.

CVD, cardiovascular disease; M, month; W, week.

### Secondary outcomes

See *Table 2* and Supplementary data online, *Table S4* for a summary of findings for the non-meta-analysable studies and *Table 3* for the meta-analysed studies.

**Table 3:** A summary of findings for results of meta-analysed studies for the other secondary outcomes

	Time point	Total number of studies	Primary prevention		Secondary prevention	Pooled analysis	Figure number in the supplement
			With usual care control	With active control	With usual care control		
**Psychological response**							
Decisional conflict score	Immediate	9	**MD −5.50 (95% CI** = **−8.58, −2.42),** ***I*^2^** = **78%**	**MD 0 (95% CI** = **−2.67, −2.67),** ***I*^2^** = **not applicable^a^**	MD −3.25 (95% CI = −8.34, 1.83), *I*^2^ = 66%	**MD −4.25 (95% CI** = **−6.58, −1.93),** ***I*^2^** = **78%**	Supplementary data online, *Figure S2*
**Smoking**							
Intention to stop	Immediate	4	OR 1.65 (95% CI = 0.96, 2.85), *I*^2^ = 0%				Supplementary data online, *Figure S3A*
Actual cessation	Up to 12 months	9	OR 1.07 (95% CI = 0.63, 1.81), *I*^2^ = 55%	OR 0.61 (95% CI = 0.26, 1.43), *I*^2^ = not applicable**^a^**	OR 1.77 (95% CI = 0.16, 19.76), *I*^2^ = 18%	OR 1 (95% CI = 0.65, 1.54), *I*^2^ = 41%	Supplementary data online, *Figure S3B*
**Physical activity**							
Intention to change	Immediate	5	OR 0.97 (95% CI = 0.81, 1.16), *I*^2^ = 73%	OR 1.43 (95% CI = 0.88, 2.34), *I*^2^ = not applicable^a^		OR 1.02 (95% CI = 0.86, 1.21), *I*^2^ = 69%	Supplementary data online, *Figure S4A*
Actual change	Up to 12 months	9	OR 1.09 (95% CI = 0.85, 1.39), *I*^2^ = 0%	OR 0.94 (95% CI = 0.73, 1.21), *I*^2^ = 0%	OR 1.18 (95% CI = 0.80, 1.73), *I*^2^ = 45%	OR 1.05 (95% CI = 0.91, 1.21), *I*^2^ = 0%	Supplementary data online, *Figure S4B*
**Diet**							
Intention to change	Immediate	4	OR 0.71 (95% CI = 0.38, 1.34), *I*^2^ = 82%				Supplementary data online, *Figure S5A*
Actual change	Up to 12 months	6	**OR 1.76 (95% CI** = **1.39, 2.22),** ***I*^2^** = **3%**	OR 1.35 (95% CI = 0.94, 1.94), *I*^2^ = 0%	OR 1.10 (95% CI = 0.65, 1.87), *I*^2^ = 24%	**OR 1.50 (95% CI** = **1.21, 1.86),** ***I*^2^** = **29%**	Supplementary data online, *Figure S5B*
**Medication adherence**							
Intention to change	Immediate	7	**OR 1.37 (95% CI** = **1.15, 1.62),** ***I*^2^** = **81%**	OR 1 (95% CI = 0.85, 1.17), *I*^2^ = not applicable**^a^**	**OR 1.44 (95% CI** = **1.15, 1.80),** ***I*^2^** = **not applicable^a^**	**OR 1.21 (95% CI** = **1.09, 1.34),** ***I*^2^** = **80%**	Supplementary data online, *Figure S6A*
Actual change	Up to 12 months	11	**OR 1.82 (95% CI** = **1.14, 2.93),** ***I*^2^** = **52%**		OR 1.22 (95% CI 0.95, 1.56), *I*^2^ = 29%	OR 1.45 (95% CI = 1, 2.11), *I*^2^ = 85%	Supplementary data online, *Figure S6B*
**CVD risk factors**							
Cholesterol	Up to 12 months	20	MD −0.09 (95% CI = −0.17, 0), *I*^2^ = 76%		**MD −0.12 (95% CI** = **−0.22, −0.02),** ***I*^2^** = **80%**	**MD −0.10 (95% CI** = **−0.16, −0.03),** ***I*^2^** = **85%**	Supplementary data online, *Figure S7*
Blood glucose	Up to 12 months	7	MD 0.02 (95% CI = −0.02, 0.05), *I*^2^ = 0%		MD −0.01 (95% CI = −0.03, 0), *I*^2^ = 0%	MD −0.01 (95% CI = −0.02, 0.01), *I*^2^ = 0%	Supplementary data online, *Figure S8*
Blood pressure	Up to 12 months	23	**MD −1.36 (95% CI** = **−2.37, −0.35),** ***I*^2^** = **39%**		MD −1.67 (95% CI = −3.41, 0.07), *I*^2^ = 72%	**MD −1.67 (95% CI** = −**2.70,** −**0.63),** ***I*^2^** = **75%**	Supplementary data online, *Figure S9*
**Clinician’s Prescribing**							
	Up to 12 months	8	OR 1.05 (95% CI = 0.72, 1.55), *I*^2^ = 62%		OR 2.05 (95% CI = 1.04, 4.02), *I*^2^ = 0%	OR 1.16 (95% CI = 0.80, 1.69), *I*^2^ = 61%	Supplementary data online, *Figure S10*

^a^Only one study in the meta-analysis. Bolded values are significant.

MD, mean difference; OR, odds ratio; RR, risk ratio; 95% CI, 95% confidence interval.

#### Change in cardiovascular disease risk score

##### Meta-analysable studies

**Figure 4:**
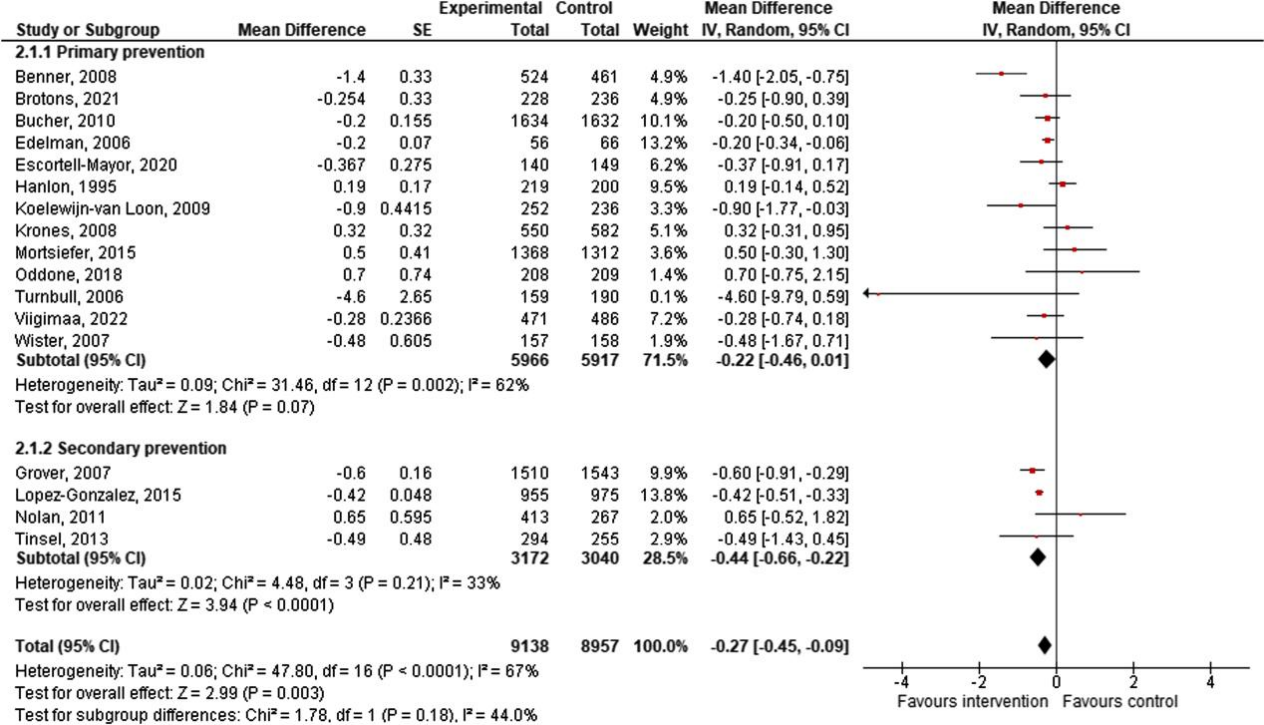
Change in cardiovascular disease risk score at 6–12 months of follow-up (*n* = 17 studies). CI, confidence interval; SE, standard error

The overall influence of CVD risk communication on the change in actual risk score at 6- to 12-month follow-up indicates a significantly larger reduction in risk for the group using CVD risk communication tools than for those receiving usual care [17 studies, pooled estimate MD = −0.27, 95% CI = −0.45, −0.09 with moderate heterogeneity (*I*^2^ = 67%); *Figure 4*]. A test for subgroup difference showed no evidence that the effect of intervention differed across subgroups (*P* = .18).

##### Non-meta-analysable studies

Two of the three interventional studies that could not be meta-analysed did not show a statistically significant effect on CVD risk score at either 3, 6, or 12 months.^43,49^ One workplace-based multi-component intervention study achieved a small but significant 12-month difference in risk score (−1.33 or 22.6%, *P* = .013) among people with pre-existing CVD^52^ (*Table 2* and Supplementary data online, *Table S4*).

#### Psychological response

Meta-analysis of nine studies showed a reduction of decisional conflict at the primary follow-up favouring CVD risk communication (MD = −4.25, 95% CI = −6.58, −1.93) with high heterogeneity (*I*^2^ = 78%; Supplementary data online, *Figure S2*). A test for subgroup difference showed evidence that the effect of intervention differed across subgroups (*P* = .03).

#### Smoking cessation intention and reported change

Meta-analysis was possible among the more homogeneous outcomes illustrating participant-reported short-term intention to change behaviour (e.g. smoking cessation, exercise, diet, and start medications). Although people without established disease exposed to the CVD risk communication appeared to have an increased intention to quit smoking, there was no significant difference compared with people in the usual care group in the four small studies that measured this outcome (OR = 1.65, 95% CI = 0.96, 2.85; Supplementary data online, *Figure S3A*).

At follow-up, no significant impact of CVD risk communication on actual smoking cessation was observed when pooling studies in primary or secondary prevention with active control groups or usual care, with no overall significant impact of CVD risk communication (OR = 1.00, 95% CI = 0.65, 1.54, *I*^2^ = 41%; Supplementary data online, *Figure S3B*).

#### Physical activity intention and reported change

Meta-analysis results suggest that cardiovascular risk communication does not enhance patients’ intention to change physical activity (pooled effect for four studies; OR = 1.02, 95% CI = 0.86, 1.21; Supplementary data online, *Figure S4A*). Likewise, there was no statistically significant impact of CVD risk communication on self-reported change in physical activity at follow-up for either primary or secondary prevention patients (pooled effect for nine studies; OR = 1.05, 95% CI = 0.91, 1.21; Supplementary data online, *Figure S4B*).

#### Dietary modifications intention and reported change

Four primary prevention studies reported patient’s intention to change their diet after CVD risk communication and showed no difference between intervention and usual care (OR = 0.71, 95% CI = 0.38, 1.34; Supplementary data online, *Figure S5A*).

At follow-up, the pooled estimate of self-reported dietary changes in seven studies indicated a statistically significant impact of the CVD risk communication in this primary prevention target group when compared with usual care (OR = 1.76, 95% CI = 1.39, 2.22; Supplementary data online, *Figure S5B*). An overall improvement in self-reported dietary change after CVD risk communication was observed for either primary or secondary prevention patients (seven studies of low heterogeneity *I*^2^ = 29%, OR = 1.50, 95% CI = 1.21, 1.86; Supplementary data online, *Figure S5B*).

#### Impact on medication intention and reported change

Compared with usual care, the impact of CVD risk communication on patient’s intention to initiate, switch, increase, or adhere to preventive medication (such as cholesterol or blood pressure lowering tablets or aspirin) was statistically significantly better for people in the intervention groups with and without established disease (seven studies, OR = 1.21, 95% CI = 1.09, 1.34; Supplementary data online, *Figure S6A*). This direction of effect remained significant for actual initiation or change in medication at follow-up for the primary prevention interventions with usual care (five studies of moderate heterogeneity *I*^2^ = 52%, OR = 1.82, 95% CI = 1.14, 2.93; Supplementary data online, *Figure S6B*).

Among the non-meta-analysable studies in *Table 2*, the intention to start any preventive medication did not significantly change within the intervention group for people without established disease. The interventions did not modify actual medication adherence at any follow-up time between 6 and 18 months either.

#### Impact on cardiovascular disease risk factors

##### Cholesterol

The overall impact of CVD risk communication on measured cholesterol levels was a small but statistically significant reduction at follow-up (20 studies with high heterogeneity *I*^2^ = 85%, MD = −0.10 mmol/L, 95% CI = −0.16, −0.03; Supplementary data online, *Figure S7*), with no significant subgroup differences (*P* = .64).

##### Blood glucose

By contrast, regardless of study sample size or the presence or absence of CVD, there was no difference in the follow-up blood glucose levels between those exposed to their CVD risk at baseline and those receiving usual care in the seven studies that reported it (pooled MD = −0.01 mmol/L, 95% CI = −0.02, 0.01; Supplementary data online, *Figure S8*).

##### Blood pressure

Cardiovascular disease risk communication delivered either face to face or remotely via the web led to a small but statistically significant reduction in mean blood pressure at follow-up (23 studies, MD = −1.67 mmHg, 95% CI = −2.70, −0.63; Supplementary data online, *Figure S9*).

#### Impact on clinician’s prescribing

None of the primary or secondary prevention interventions using CVD risk communication led to significantly higher rates in clinician prescription of medication to reduce the risk factors identified than those in the control groups (seven studies, pooled OR = 1.16, 95% CI = 0.80, 1.69; Supplementary data online, *Figure S10*). With one exception,^64^ these trials of actual prescribing reported by clinicians were not the same as those studies where patients reported medication use, so discrepancies in reporting between patients and clinicians could not be examined.

## Discussion

### Summary of findings

This systematic review of 62 trials of generally low risk of bias found that disclosing and communicating cardiovascular risk had a mixed impact on intention to change and subsequent behaviours (*Structured Graphical Abstract*). Not all studies reported all outcomes of interest and not all outcomes were amenable to meta-analyses. However, to our knowledge, this is the first systematic review investigating the impact on both patients and clinicians.

Overall, and relying largely on the studies that were meta-analysable, the communication of CVD risk led to an increased accuracy of risk perception that was two- to three-fold higher than the control groups, with a greater effect on patients without established CVD (nine studies). Risk communication also led to small but significant reductions in the cardiovascular risk score at 6- to 12-month follow-up (17 studies). It is possible that most of this change was derived from the increase in initiation or adherence to preventive medications (11 studies) or the reported changes in dietary behaviours (7 studies).

Further objective benefits of CVD risk communication were reductions in mean blood pressure at follow-up (23 studies) and blood cholesterol (20 studies).

From the behavioural perspective, intentions to stop smoking or modify physical activity after exposure to risk communication were not different to those of people in the control group; and self-reported smoking cessation or actual changes in physical activity at follow-up did not differ between active and control groups for patients with or without established CVD. Cardiovascular disease risk communication interventions had no impact on the intention to modify diet among patients without established CVD (four studies). Yet, it significantly improved dietary changes in primary prevention interventions when compared with usual care (four studies) and overall, despite not having an impact on secondary prevention (two studies) or primary prevention against active control (two studies). Knowing CVD risk increased patients’ intention to initiate or adhere to medication for primary and secondary prevention patients (five studies) and actual medication adherence at follow-up (11 studies). This suggests that people prefer to rely on pharmacotherapy to reduce cardiovascular risk than engage in proactive goal setting, self-regulation, and self-monitoring efforts^87^ to modify harder-to-achieve lifestyle behaviours such as physical activity and smoking cessation. The impact of the unreliable nature of self-reported outcomes cannot be discounted as a reason for the lack of success in these interventions. Yet, it did not change clinician’s preventive prescribing behaviour (seven studies). While this is a disappointing finding, clinical decision-making may have incorporated patient preference or other risks such as side effects or concurrent co-morbidities, as recommended in the guidelines.^88^

Findings from the non-meta-analysable studies were also mixed, with the most impact on positive psychological response (five studies), accuracy of risk perception (three studies), and self-reported smoking cessation for people without established disease (four studies), and somewhat on physical activity and diet (two studies each).

### Results in context with other literature

Non-RCT study designs have reported high levels of inaccuracy of risk perception. A before–after study of adult (47 ± 15 years) community dwellers in the USA reported 66% baseline inaccuracy in relation to their objective Framingham risk calculation. The discrepancy was mostly underestimation of 5-year CVD risk (low, moderate, and high) among non-Africans aged >45 years who used alcohol.^89^

Large prospective initiatives continue to promote the use of risk assessment tools for periodic monitoring to inform personalized therapeutic decisions among adults at risk.^90^ Further, while closing the gap between known cardiovascular risk and lifestyle behaviour change remains a challenge,^91–93^ the importance of identifying and communicating prognostic cardiovascular risk cannot be underestimated. Not only it is known to predict disability and mortality,^94^ but also associations between cardiovascular risk factors and cognitive impairment have been reported from large cohort studies.^95,96^ There is some evidence that small efforts to minimize several risk factors may prove as beneficial or more effective than substantial reduction in a single risk factor.^97^ Ways to sustain the change over time include multi-disciplinary team engagement, and consideration of the social determinants of health,^97^ to determine level of support required.

The impact of CVD risk communication is also known to vary according to the type of risk format used, although our analysis did not focus on this. Of note, the diversity of ways in which risk was communicated in these studies is part of real-life practice where approaches vary depending on provider preference, patient literacy, available evidence, or local health service policy.

Following the completion of our review, a 2022 systematic review of nine tools of diverse formats reported the biggest impact on both intention and actual behaviour derived from tools that used CVD imaging.^98^ A recent review of quantitative and qualitative studies investigated the impact of the *Heart Age* CVD risk tool on psychological, behavioural, and clinical outcomes when compared with absolute risk calculation. The authors reported enhanced psychological responses and increased risk perception with *Heart Age* but no impact on intention to pursue lifestyle modifications.^99^ By contrast, the *Fitness Age* tool, was associated with reduced intentions to change lifestyle when compared with *Heart Age*. A simulation study of 25 risk calculators generating risk categories low, moderate, or high based on seven risk factors found the risk estimates to be non-reproducible for different timeframes (5- or 10-year risk) and patient types (e.g. diabetes vs. not). The authors warned about using risk calculators to inform clinical practice guidelines.^100^

Despite this, CVD risk calculation and communication are currently recommended by the Australian Heart, Stroke, Diabetes, and Kidney foundations not only to make patients aware of their modifiable lifestyle factors but also for clinicians to decide on monitoring frequency and to guide medication adjustment.^101^ For doctors, best practice CVD risk communication should include assessment of modifiable and not modifiable risk factors and consideration of family and personal history of comorbidities. However, management decisions to reduce risk will need to adapt a patient-centred approach^102^ with balanced discussion on risk and benefits of pharmacotherapy, impact on quality of life, physical and financial burden of treatment, monitoring demands, and personal preferences.

### Limitations of this review

Our intention to document multiple outcomes was driven by the need to investigate impacts on both patients and clinicians’ behaviour to capture the overall effects. However, many of the outcomes were self-reported and, hence, selected results need to be viewed with caution. Although there is evidence that self-report of own risk factors is accurate for risk calculation,^103^ the reliability and accuracy of patient-reported/self-report of risk perception and health behaviours such as lifestyle modifications and medication adherence may be flawed with over- or under-estimations related to recall,^104^ health literacy, single/multiple-item survey instrument, or to satisfy social desirability.^105–107^ Some potentially eligible studies may have been excluded if the full text did not clearly state that the measured CVD risk was communicated to participants. For the primary outcome, the accuracy of risk perception was assessed differently among the included studies, which could have contributed to the high heterogeneity reported in the analysis. There were multiple eligible studies not amenable to inclusion in the meta-analysis due to heterogeneity of samples, interventions, and outcome measurements. Their results, presented in the supplement, need to be viewed with caution.

Many of the interventions in this review were multi-component, and hence, it is acknowledged that this resource-intensive investment for wider implementation in routine care may not be feasible.

### Implications for practice and research

As CVD risk communication increases risk perception and leads to quantifiable overall risk reduction, it is recommended for clinicians to continue communicating CVD risk with or without decision support tools, particularly for people without an established disease where the impact appears more widespread across risk factor modification. Caution is recommended in the potential for overestimation of risk from tools calibrated for populations from other countries and the variation in patient’s willingness to start pharmacotherapy to reduce cardiovascular events after becoming aware of their risk.^108^ Another important consideration is the need to further investigate whether reasons for the apparent lack of response from clinicians in increasing prescribing of preventative medications to reduce CVD risk were consumer or provider driven.

## Conclusions

This systematic review revealed that CVD risk communication has a mixed impact on intentions and behavioural change for different risk factors. Disclosing and communicating cardiovascular risk levels to at-risk patients has a favourable effect on enhancing accuracy and awareness of self-perceived risk and lowers overall risk score after 6–12 months of follow-up, as well as blood pressure and cholesterol levels. This effect was greater for adults without established CVD. Motivation intentions and actual change in smoking or physical activity were not significantly impacted by CVD risk communications among people with or without established disease. However, actual change in diet was significant at follow-up for the primary prevention participants. Patient-reported intention to medication commencement or ongoing adherence was significantly improved by CVD risk communication in both primary and secondary prevention interventions with actual changes reported in the primary prevention group, but clinicians’ change in preventive prescribing behaviour did not align with this finding, possibly due to genuine patient factors. We conclude that it is worth making patients aware of their risk levels to achieve some gain in overall risk reduction regardless of the individual risk factor impacted.

## Supplementary Material

ehae002_Supplementary_Data

## Data Availability

The data underlying this article are available in the article and in its online Supplementary material.

## Pre-registered Clinical Trial Number

None supplied.

## References

[1] World Health Organization . 2021. *Health Topics: Cardiovascular Diseases Factsheet*. https://www.who.int/health-topics/cardiovascular-diseases#tab=tab_1.

[2] Australian Institute of Health and Welfare . 2021. *Heart, Stroke and Vascular Disease—Australian Facts*. https://www.aihw.gov.au/reports/heart-stroke-vascular-diseases/hsvd-facts/contents/about.

[3] Schwalm JD , McKee M, Huffman MD, Yusuf S. Resource effective strategies to prevent and treat cardiovascular disease. Circulation 2016;133:742–55. 10.1161/CIRCULATIONAHA.115.008721 26903017 PMC4766731

[4] Brunner E , Cohen D, Toon L. Cost effectiveness of cardiovascular disease prevention strategies: a perspective on EU food based dietary guidelines. Public Health Nutr 2001;4:711–5. 10.1079/PHN2001161 11683566

[5] Rippe JM . Lifestyle strategies for risk factor reduction, prevention, and treatment of cardiovascular disease. Am J Lifestyle Med 2019;13:204–12. 10.1177/1559827618812395 30800027 PMC6378495

[6] Navar AM , Wang TY, Mi X, Robinson JG, Virani SS, Roger VL, et al Influence of cardiovascular risk communication tools and presentation formats on patient perceptions and preferences. JAMA Cardiol 2018;3:1192–9. 10.1001/jamacardio.2018.3680 30419113 PMC6551302

[7] Lipkus IM , Samsa G, Rimer BK. General performance on a numeracy scale among highly educated samples. Med Decis Making 2001;21:37–44. 10.1177/0272989X0102100105 11206945

[8] Michie S , West R, Sheals K, Godinho CA. Evaluating the effectiveness of behavior change techniques in health-related behavior: a scoping review of methods used. Transl Behav Med 2018;8:212–24. 10.1093/tbm/ibx019 29381786 PMC6062857

[9] Schroé H , Van Dyck D, De Paepe A, Poppe L, Loh WW, Verloigne M, et al Which behaviour change techniques are effective to promote physical activity and reduce sedentary behaviour in adults: a factorial randomized trial of an e- and m-health intervention. Int J Behav Nutr Phys Act 2020;17:127. 10.1186/s12966-020-01001-x 33028335 PMC7539442

[10] Sheridan SL , Viera AJ, Krantz MJ, Ice CL, Steinman LE, Peters KE, et al The effect of giving global coronary risk information to adults: a systematic review. Arch Intern Med 2010;170:230–9. 10.1001/archinternmed.2009.516 20142567

[11] Bonner C , Fajardo MA, Hui S, Stubbs R, Trevena L. Clinical validity, understandability, and actionability of online cardiovascular disease risk calculators: systematic review. J Med Internet Res 2018;20:e29. 10.2196/jmir.8538 29391344 PMC5814602

[12] Studziński K , Tomasik T, Krzysztoń J, Jóźwiak J, Windak A. Effect of using cardiovascular risk scoring in routine risk assessment in primary prevention of cardiovascular disease: an overview of systematic reviews. BMC Cardiovasc Disord 2019;19:11. 10.1186/s12872-018-0990-2 30626326 PMC6327540

[13] Page MJ , McKenzie JE, Bossuyt PM, Boutron I, Hoffmann TC, Mulrow CD, et al The PRISMA 2020 statement: an updated guideline for reporting systematic reviews. BMJ 2021;372:n71. 10.1136/bmj.n71 33782057 PMC8005924

[14] Hogg RV , Tanis EA, Zimmerman DL. Probability and Statistical Inference. Upper Saddle River, NJ, USA: Pearson/Prentice Hall, 2010.

[15] Clark JM , Sanders S, Carter M, Honeyman D, Cleo G, Auld Y, et al Improving the translation of search strategies using the Polyglot Search Translator: a randomized controlled trial. J Med Libr Assoc 2020;108:195–207. 10.5195/jmla.2020.834 32256231 PMC7069833

[16] Clark J , Glasziou P, Del Mar C, Bannach-Brown A, Stehlik P, Scott AM. A full systematic review was completed in 2 weeks using automation tools: a case study. J Clin Epidemiol 2020;121:81–90. 10.1016/j.jclinepi.2020.01.008 32004673

[17] Hoffmann TC , Glasziou PP, Boutron I, Milne R, Perera R, Moher D, et al Better reporting of interventions: template for intervention description and replication (TIDieR) checklist and guide. BMJ 2014;348:g1687. 10.1136/bmj.g1687 24609605

[18] Higgins JPT , Altman DG, Gøtzsche PC, Jüni P, Moher D, Oxman AD, et al The cochrane collaboration’s tool for assessing risk of bias in randomised trials. BMJ 2011;343:d5928. 10.1136/bmj.d5928 22008217 PMC3196245

[19] Campbell M , McKenzie JE, Sowden A, Katikireddi SV, Brennan SE, Ellis S, et al Synthesis without meta-analysis (SWiM) in systematic reviews: reporting guideline. BMJ 2020;368:l6890. 10.1136/bmj.l6890 31948937 PMC7190266

[20] Wood DA , Kinmonth AL, Davies GA, Yarwood J, Thompson SG, Pyke SDM, et al Family Heart Study Group. Randomised controlled trial evaluating cardiovascular screening and intervention in general practice: principal results of British family heart study. BMJ 1994;308:313–20. 10.1136/bmj.308.6924.313 8124121 PMC2539278

[21] Adarkwah CC , Jegan N, Heinzel-Gutenbrunner M, Kuhne F, Siebert U, Popert U, et al The Optimizing–risk–communication (OptRisk) randomized trial—impact of decision-aid-based consultation on adherence and perception of cardiovascular risk. Patient Prefer Adherence 2019;13:441–52. 10.2147/PPA.S197545 30988601 PMC6441552

[22] Avis NE , Smith KW, McKinlay JB. Accuracy of perceptions of heart attack risk: what influences perceptions and can they be changed? Am J Public Health 1989;79:1608–12. 10.2105/AJPH.79.12.1608 2817187 PMC1349762

[23] Ayres K , Conner M, Prestwich A, Hurling R, Cobain M, Lawton R, et al Exploring the question-behaviour effect: randomized controlled trial of motivational and question-behaviour interventions. Br J Health Psychol 2013;18:31–44. 10.1111/j.2044-8287.2012.02075.x 22519696

[24] Benner JS , Erhardt L, Flammer M, Moller RA, Rajicic N, Changela K, et al A novel programme to evaluate and communicate 10-year risk of CHD reduces predicted risk and improves patients’ modifiable risk factor profile. Int J Clin Pract 2008;62:1484–98. 10.1111/j.1742-1241.2008.01872.x 18691228 PMC2658032

[25] Bonner C , Jansen J, Newell BR, Irwig L, Teixeira-Pinto A, Glasziou P, et al Is the “heart age” concept helpful or harmful compared to absolute cardiovascular disease risk? An experimental study. Med Decis Making 2015;35:967–78. 10.1177/0272989X15597224 26251465

[26] Broadbent E , Leggat A, McLachlan A, Kerr A. Providing cardiovascular risk management information to acute coronary syndrome patients: a randomized trial. Br J Health Psychol 2013;18:83–96. 10.1111/j.2044-8287.2012.02081.x 22709363

[27] Brotons C , Moral I, Fernandez D, Puig M, Vilella MT, Puig T, et al Effectiveness of an intervention aimed at improving information for patients with high cardiovascular risk: INFORISK clinical trial. Int J Environ Res Public Health 2021;18:3621. 10.3390/ijerph18073621 33807285 PMC8036291

[28] Bucher HC , Rickenbach M, Young J, Glass TR, Vallet Y, Bernasconi E, et al Randomized trial of a computerized coronary heart disease risk assessment tool in HIV-infected patients receiving combination antiretroviral therapy. Antivir Ther 2010;15:31–40. 10.3851/IMP1475 20167989

[29] Buhse S , Kuniss N, Liethmann K, Muller UA, Lehmann T, Muhlhauser I. Informed shared decision-making programme for patients with type 2 diabetes in primary care: cluster randomised controlled trial. BMJ Open 2018;8:e024004. 10.1136/bmjopen-2018-024004 PMC6303685 30552272

[30] Buhse S , Muhlhauser I, Heller T, Kuniss N, Muller UA, Kasper J, et al Informed shared decision-making programme on the prevention of myocardial infarction in type 2 diabetes: a randomised controlled trial. BMJ Open 2015;5:e009116. 10.1136/bmjopen-2015-009116 PMC4654390 26567256

[31] Christensen B , Engberg M, Lauritzen T. No long-term psychological reaction to information about increased risk of coronary heart disease in general practice. Eur J Cardiovasc Prev Rehabil 2004;11:239–43. 10.1097/01.hjr.0000129739.30593.23 15179107

[32] Cioe PA , Merrill JE, Gordon REF, Guthrie KM, Freiberg M, Williams DM, et al Personalized feedback improves cardiovascular risk perception and physical activity levels in persons with HIV: results of a pilot randomized clinical trial. AIDS Care 2021;33:786–94. 10.1080/09540121.2021.1874271 33486982 PMC8300575

[33] Edelman D , Oddone EZ, Liebowitz RS, Yancy WS Jr, Olsen MK, Jeffreys AS, et al A multidimensional integrative medicine intervention to improve cardiovascular risk. J Gen Intern Med 2006;21:728–34. 10.1111/j.1525-1497.2006.00495.x 16808774 PMC1924710

[34] Escortell-Mayor E , Del Cura-Gonzalez I, Ojeda-Ruiz E, Sanz-Cuesta T, Rodriguez-Salceda I, Garcia-Soltero J, et al A primary healthcare information intervention for communicating cardiovascular risk to patients with poorly controlled hypertension: the Education and Coronary Risk Evaluation (Educore) study—a pragmatic, cluster-randomized trial. PLoS One 2020;15:e0226398. 10.1371/journal.pone.0226398 31971952 PMC6977759

[35] Fernandez RS , Davidson P, Griffiths R, Juergens C, Stafford B, Salamonson Y. A pilot randomised controlled trial comparing a health-related lifestyle self-management intervention with standard cardiac rehabilitation following an acute cardiac event: implications for a larger clinical trial. Aust Crit Care 2009;22:17–27. 10.1016/j.aucc.2008.10.003 19081265

[36] Grover SA , Lowensteyn I, Joseph L, Kaouache M, Marchand S, Coupal L, et al Discussing coronary risk with patients to improve blood pressure treatment: secondary results from the CHECK-UP study. J Gen Intern Med 2009;24:33–9. 10.1007/s11606-008-0825-4 18937013 PMC2607501

[37] Grover SA , Lowensteyn I, Joseph L, Kaouache M, Marchand S, Coupal L, et al Patient knowledge of coronary risk profile improves the effectiveness of dyslipidemia therapy: the CHECK-UP study: a randomized controlled trial. Arch Intern Med 2007;167:2296–303. 10.1001/archinte.167.21.2296 18039987

[38] Hanlon P , McEwen J, Carey L, Gilmour H, Tannahill C, Tannahill A, et al Health checks and coronary risk: further evidence from a randomised controlled trial. BMJ 1995;311:1609–13. 10.1136/bmj.311.7020.1609 8555805 PMC2551502

[39] Hess EP , Hollander JE, Schaffer JT, Kline JA, Torres CA, Diercks DB, et al Shared decision making in patients with low risk chest pain: prospective randomized pragmatic trial. BMJ 2016;355:i6165. 10.1136/bmj.i6165 27919865 PMC5152707

[40] Hess EP , Knoedler MA, Shah ND, Kline JA, Breslin M, Branda ME, et al The chest pain choice decision aid: a randomized trial. Circ Cardiovasc Qual Outcomes 2012;5:251–9. 10.1161/CIRCOUTCOMES.111.964791 22496116

[41] Jacobson TA , Gutkin SW, Harper CR. Effects of a global risk educational tool on primary coronary prevention: the Atherosclerosis Assessment Via Total Risk (AVIATOR) study. Curr Med Res Opin 2006;22:1065–73. 10.1185/030079906X104605 16846539

[42] Jaspers NEM , Visseren FLJ, van der Graaf Y, Smulders YM, Damman OC, Brouwers C, et al Communicating personalised statin therapy-effects as 10-year CVD-risk or CVD-free life-expectancy: does it improve decisional conflict? Three-armed, blinded, randomised controlled trial. BMJ Open 2021;11:e041673. 10.1136/bmjopen-2020-041673 PMC8287608 34272216

[43] Kask-Flight L , Durak K, Suija K, Ratsep A, Kalda R. Reduction of cardiovascular risk factors among young men with hypertension using an interactive decision aid: cluster-randomized control trial. BMC Cardiovasc Disord 2021;21:543. 10.1186/s12872-021-02339-1 34784891 PMC8596802

[44] Koelewijn-van Loon MS , van der Weijden T, Ronda G, van Steenkiste B, Winkens B, Elwyn G, et al Improving lifestyle and risk perception through patient involvement in nurse-led cardiovascular risk management: a cluster-randomized controlled trial in primary care. Prev Med 2010;50:35–44. 10.1016/j.ypmed.2009.11.007 19944713

[45] Koelewijn-van Loon MS , van der Weijden T, van Steenkiste B, Ronda G, Winkens B, Severens JL, et al Involving patients in cardiovascular risk management with nurse-led clinics: a cluster randomized controlled trial. CMAJ 2009;181:E267–74. 10.1503/cmaj.081591 19948811 PMC2789146

[46] Krones T , Keller H, Sonnichsen A, Sadowski EM, Baum E, Wegscheider K, et al Absolute cardiovascular disease risk and shared decision making in primary care: a randomized controlled trial. Ann Fam Med 2008;6:218–27. 10.1370/afm.854 18474884 PMC2384995

[47] Kunneman M , Branda ME, Hargraves IG, Sivly AL, Lee AT, Gorr H, et al Assessment of shared decision-making for stroke prevention in patients with atrial fibrillation: a randomized clinical trial. JAMA Intern Med 2020;180:1215–24. 10.1001/jamainternmed.2020.2908 32897386 PMC7372497

[48] Lopez-Gonzalez AA , Aguilo A, Frontera M, Bennasar-Veny M, Campos I, Vicente-Herrero T, et al Effectiveness of the Heart Age tool for improving modifiable cardiovascular risk factors in a Southern European population: a randomized trial. Eur J Prev Cardiol 2015;22:389–96. 10.1177/2047487313518479 24491403

[49] Lovibond SH , Birrell PC, Langeluddecke P. Changing coronary heart disease risk-factor status: the effects of three behavioral programs. J Behav Med 1986;9:415–37. 10.1007/BF00845131 3540308

[50] Lowensteyn I , Joseph L, Levinton C, Abrahamowicz M, Steinert Y, Grover S. Can computerized risk profiles help patients improve their coronary risk? The results of the Coronary Health Assessment Study (CHAS). Prev Med 1998;27:730–7. 10.1006/pmed.1998.0351 9808805

[51] Mann DM , Ponieman D, Montori VM, Arciniega J, McGinn T. The Statin Choice decision aid in primary care: a randomized trial. Patient Educ Couns 2010;80:138–40. 10.1016/j.pec.2009.10.008 19959322

[52] Maron DJ , Forbes BL, Groves JR, Dietrich MS, Sells P, DiGenio AG. Health-risk appraisal with or without disease management for worksite cardiovascular risk reduction. J Cardiovasc Nurs 2008;23:513–8. 10.1097/01.JCN.0000338933.81587.b4 18953215

[53] Marteau TM , Kinmonth AL, Thompson S, Pyke S. The psychological impact of cardiovascular screening and intervention in primary care: a problem of false reassurance? British Family Heart Study Group. Br J Gen Pract 1996;46:577–82. https://bjgp.org/content/46/411/577 8945794 PMC1239781

[54] Mitchell E , Sullivan F, Grimshaw JM, Donnan PT, Watt G. Improving management of hypertension in general practice: a randomised controlled trial of feedback derived from electronic patient data. Br J Gen Pract 2005;55:94–101. https://bjgp.org/content/55/511/94.long 15720929 PMC1463214

[55] Montgomery AA , Fahey T, Peters TJ. A factorial randomised controlled trial of decision analysis and an information video plus leaflet for newly diagnosed hypertensive patients. Br J Gen Pract 2003;53:446–53. https://www.ncbi.nlm.nih.gov/pmc/articles/PMC1314618/12939889 PMC1314618

[56] Montgomery AA , Fahey T, Peters TJ, MacIntosh C, Sharp DJ. Evaluation of computer based clinical decision support system and risk chart for management of hypertension in primary care: randomised controlled trial. BMJ 2000;320:686–90. 10.1136/bmj.320.7236.686 10710578 PMC27312

[57] Mortsiefer A , Meysen T, Schumacher M, Abholz HH, Wegscheider K, In der Schmitten J. From hypertension control to global cardiovascular risk management: an educational intervention in a cluster-randomised controlled trial. BMC Fam Pract 2015;16:56. 10.1186/s12875-015-0274-1 25947301 PMC4426642

[58] Nieuwkerk PT , Nierman MC, Vissers MN, Locadia M, Greggers-Peusch P, Knape LP, et al Intervention to improve adherence to lipid-lowering medication and lipid-levels in patients with an increased cardiovascular risk. Am J Cardiol 2012;110:666–72. 10.1016/j.amjcard.2012.04.045 22621795

[59] Nolan RP , Upshur RE, Lynn H, Crichton T, Rukholm E, Stewart DE, et al Therapeutic benefit of preventive telehealth counseling in the Community Outreach Heart Health and Risk Reduction Trial. Am J Cardiol 2011;107:690–6. 10.1016/j.amjcard.2010.10.050 21215382

[60] Oddone EZ , Gierisch JM, Sanders LL, Fagerlin A, Sparks J, McCant F, et al A coaching by telephone intervention on engaging patients to address modifiable cardiovascular risk factors: a randomized controlled trial. J Gen Intern Med 2018;33:1487–94. 10.1007/s11606-018-4398-6 29736750 PMC6108991

[61] Peiris D , Usherwood T, Panaretto K, Harris M, Hunt J, Patel B, et al The Treatment of cardiovascular Risk in Primary care using Electronic Decision supOrt (TORPEDO) study-intervention development and protocol for a cluster randomised, controlled trial of an electronic decision support and quality improvement intervention in Australian primary healthcare. BMJ Open 2012;2:e002177. 10.1136/bmjopen-2012-002177 PMC3533097 23166140

[62] Peiris D , Usherwood T, Panaretto K, Harris M, Hunt J, Redfern J, et al Effect of a computer-guided, quality improvement program for cardiovascular disease risk management in primary health care the treatment of cardiovascular risk using electronic decision support cluster-randomized trial. Circ-Cardiovasc Qual 2015;8:87–95. 10.1161/CIRCOUTCOMES.114.001235 25587090

[63] Perestelo-Perez L , Rivero-Santana A, Boronat M, Sanchez-Afonso JA, Perez-Ramos J, Montori VM, et al Effect of the statin choice encounter decision aid in Spanish patients with type 2 diabetes: a randomized trial. Patient Educ Couns 2016;99:295–9. 10.1016/j.pec.2015.08.032 26343571

[64] Persell SD , Brown T, Lee JY, Shah S, Henley E, Long T, et al Individualized risk communication and outreach for primary cardiovascular disease prevention in community health centers: randomized trial. Circ Cardiovasc Qual Outcomes 2015;8:560–6. 10.1161/CIRCOUTCOMES.115.001723 26555123

[65] Persell SD , Lloyd-Jones DM, Friesema EM, Cooper AJ, Baker DW. Electronic health record-based patient identification and individualized mailed outreach for primary cardiovascular disease prevention: a cluster randomized trial. J Gen Intern Med 2013;28:554–60. 10.1007/s11606-012-2268-1 23143672 PMC3599027

[66] Powers BJ , Danus S, Grubber JM, Olsen MK, Oddone EZ, Bosworth HB. The effectiveness of personalized coronary heart disease and stroke risk communication. Am Heart J 2011;161:673–80. 10.1016/j.ahj.2010.12.021 21473965

[67] Redfern J , Coorey G, Mulley J, Scaria A, Neubeck L, Hafiz N, et al A digital health intervention for cardiovascular disease management in primary care (CONNECT) randomized controlled trial. NPJ Digit Med 2020;3:117. 10.1038/s41746-020-00325-z 32964140 PMC7484809

[68] Sheridan SL , Draeger LB, Pignone MP, Rimer B, Bangdiwala SI, Cai J, et al The effect of a decision aid intervention on decision making about coronary heart disease risk reduction: secondary analyses of a randomized trial. BMC Med Inform Decis Mak 2014;14:14. 10.1186/1472-6947-14-14 24575882 PMC3943405

[69] Sheridan SL , Shadle J, Simpson RJ Jr, Pignone MP. The impact of a decision aid about heart disease prevention on patients’ discussions with their doctor and their plans for prevention: a pilot randomized trial. BMC Health Serv Res 2006;6:121. 10.1186/1472-6963-6-121 17005051 PMC1626460

[70] Silarova B , Sharp S, Usher-Smith JA, Lucas J, Payne RA, Shefer G, et al Effect of communicating phenotypic and genetic risk of coronary heart disease alongside web-based lifestyle advice: the INFORM randomised controlled trial. Heart 2019;105:982–9. 10.1136/heartjnl-2018-314211 30928969 PMC6582721

[71] Soureti A , Hurling R, Murray P, van Mechelen W, Cobain M. Evaluation of a cardiovascular disease risk assessment tool for the promotion of healthier lifestyles. Eur J Cardiovasc Prev Rehabil 2010;17:519–23. 10.1097/HJR.0b013e328337ccd3 20195154

[72] Soureti A , Murray P, Cobain M, van Mechelen W, Hurling R. Web-based risk communication and planning in an obese population: exploratory study. J Med Internet Res 2011;13:e100. 10.2196/jmir.1579 22126827 PMC3278086

[73] Svendsen K , Jacobs DR, Morch-Reiersen LT, Garstad KW, Henriksen HB, Telle-Hansen VH, et al Evaluating the use of the heart age tool in community pharmacies: a 4-week cluster-randomized controlled trial. Eur J Public Health 2020;30:1139–45. 10.1093/eurpub/ckaa048 32206810

[74] Svendsen K , Telle-Hansen VH, Morch-Reiersen LT, Garstad KW, Thyholt K, Granlund L, et al A randomized controlled trial in Norwegian pharmacies on effects of risk alert and advice in people with elevated cardiovascular risk. Prev Med Rep 2018;12:79–86. 10.1016/j.pmedr.2018.08.004 30191097 PMC6125803

[75] Taksler GB , Hu B, DeGrandis F Jr, Montori VM, Fagerlin A, Nagykaldi Z, et al Effect of individualized preventive care recommendations vs usual care on patient interest and use of recommendations: a pilot randomized clinical trial. JAMA Netw Open 2021;4:e2131455. 10.1001/jamanetworkopen.2021.31455 34726747 PMC8564576

[76] Tinsel I , Buchholz A, Vach W, Siegel A, Durk T, Buchholz A, et al Shared decision-making in antihypertensive therapy: a cluster randomised controlled trial. BMC Fam Pract 2013;14:135. 10.1186/1471-2296-14-135 24024587 PMC3847233

[77] Turnbull DA , Beilby JJ, Ziaian T, Qureshi F, Nelson M, Tonkin AL, et al Disease management for hypertension—a pilot cluster randomized trial of 67 Australian general practices. Dis Manag Health Out 2006;14:27–35. 10.2165/00115677-200614010-00004

[78] van Steenkiste B , van der Weijden T, Stoffers HE, Kester AD, Timmermans DR, Grol R. Improving cardiovascular risk management: a randomized, controlled trial on the effect of a decision support tool for patients and physicians. Eur J Cardiovasc Prev Rehabil 2007;14:44–50. 10.1097/01.hjr.0000239475.71805.1e 17301626

[79] Weymiller AJ , Montori VM, Jones LA, Gafni A, Guyatt GH, Bryant SC, et al Helping patients with type 2 diabetes mellitus make treatment decisions: statin choice randomized trial. Arch Intern Med 2007;167:1076–82. 10.1001/archinte.167.10.1076 17533211

[80] Wister A , Loewen N, Kennedy-Symonds H, McGowan B, McCoy B, Singer J. One-year follow-up of a therapeutic lifestyle intervention targeting cardiovascular disease risk. CMAJ 2007;177:859–65. 10.1503/cmaj.061059 17923653 PMC1995136

[81] Zullig LL , Sanders LL, Shaw RJ, McCant F, Danus S, Bosworth HB. A randomised controlled trial of providing personalised cardiovascular risk information to modify health behaviour. J Telemed Telecare 2014;20:147–52. 10.1177/1357633X14528446 24647384

[82] Sheridan SL , Draeger LB, Pignone MP, Keyserling TC, Simpson RJ, Rimer B, et al A randomized trial of an intervention to improve use and adherence to effective coronary heart disease prevention strategies. BMC Health Serv Res 2011;11:331. 10.1186/1472-6963-11-331 22141447 PMC3268742

[83] Bonner C , Batcup C, Ayre J, Cvejic E, Trevena L, McCaffery K, et al The impact of health literacy-sensitive design and heart age in a cardiovascular disease prevention decision aid: randomized controlled trial and end-user testing. JMIR Cardio 2022;6:e34142. 10.2196/34142 35436208 PMC9055529

[84] Muscat DM , Morris GM, Bell K, Cvejic E, Smith J, Jansen J, et al Benefits and harms of hypertension and high-normal labels: a randomized experiment. Circ Cardiovasc Qual Outcomes 2021;14:E007160. 10.1161/CIRCOUTCOMES.120.007160 33813855

[85] Sarfo FS , Akinyemi JO, Obiako R, Nichols M, Fakunle AG, Adusei N, et al Effect of an educational intervention for primary stroke risk reduction in Ghana and Nigeria: pilot randomized controlled trial. Stroke 2023;54:1660–4. 10.1161/STROKEAHA.123.042618 37139815 PMC10202839

[86] Viigimaa M , Jürisson M, Pisarev H, Kalda R, Alavere H, Irs A, et al Effectiveness and feasibility of cardiovascular disease personalized prevention on high polygenic risk score subjects: a randomized controlled pilot study. Eur Heart J Open 2022;2:oeac079. 10.1093/ehjopen/oeac079 36600884 PMC9803971

[87] Suls J , Mogavero JN, Falzon L, Pescatello LS, Hennessy EA, Davidson KW. Health behaviour change in cardiovascular disease prevention and management: meta-review of behaviour change techniques to affect self-regulation. Health Psychol Rev 2020;14:43–65. 10.1080/17437199.2019.1691622 31707938 PMC7018570

[88] Visseren FLJ , Mach F, Smulders YM, Carballo D, Koskinas KC, Bäck M, et al 2021 ESC guidelines on cardiovascular disease prevention in clinical practice: developed by the Task Force for cardiovascular disease prevention in clinical practice with representatives of the European Society of Cardiology and 12 medical societies with the special contribution of the European Association of Preventive Cardiology (EAPC). Eur Heart J 2021;42:3227–337. 10.1093/eurheartj/ehab484 34458905

[89] Hussein HM , Harris-Lane P, Abdelmoula MM, Vazquez G. Accuracy of self-perception of cardiovascular risk in the community. J Vasc Interv Neurol 2008;1:106–12. https://www.ncbi.nlm.nih.gov/pmc/articles/PMC3317327/22518234 PMC3317327

[90] Lloyd-Jones DM , Huffman MD, Karmali KN, Sanghavi DM, Wright JS, Pelser C, et al Estimating longitudinal risks and benefits from cardiovascular preventive therapies among Medicare patients: the million hearts longitudinal ASCVD risk assessment tool: a special report from the American Heart Association and American College of Cardiology. Circulation 2017;135:e793–813. 10.1161/CIR.0000000000000467 27815375 PMC6027623

[91] Bergum H , Sandven I, Klemsdal TO. Long-term effects (>24 months) of multiple lifestyle intervention on major cardiovascular risk factors among high-risk subjects: a meta-analysis. BMC Cardiovasc Disord 2021;21:181. 10.1186/s12872-021-01989-5 33858345 PMC8048075

[92] Lemp JM , Nuthanapati MP, Bärnighausen TW, Vollmer S, Geldsetzer P, Jani A. Use of lifestyle interventions in primary care for individuals with newly diagnosed hypertension, hyperlipidaemia or obesity: a retrospective cohort study. J R Soc Med 2022;115:289–99. 10.1177/01410768221077381 35176215 PMC9340092

[93] van Trier TJ , Mohammadnia N, Snaterse M, Peters RJG, Jørstad HT, Bax WA. Lifestyle management to prevent atherosclerotic cardiovascular disease: evidence and challenges. Neth Heart J 2022;30:3–14. 10.1007/s12471-021-01642-y 34762283 PMC8724344

[94] Makino K , Lee S, Bae S, Chiba I, Harada K, Katayama O, et al Absolute cardiovascular disease risk assessed in old age predicts disability and mortality: a retrospective cohort study of community-dwelling older adults. J Am Heart Assoc 2021;10:e022004. 10.1161/JAHA.121.022004 34913358 PMC9075253

[95] Hua W , Hou J, Jiang T, Su B, Fu J, Sun R, et al The longitudinal association between cardiovascular risk and cognitive function in middle-aged and older adults in China: a nationally representative cohort study. Front Cardiovasc Med 2020;7:560947. 10.3389/fcvm.2020.560947 33195454 PMC7604338

[96] Olaya B , Moneta MV, Bobak M, Haro JM, Demakakos P. Cardiovascular risk factors and memory decline in middle-aged and older adults: the English Longitudinal Study of Ageing. BMC Geriatr 2019;19:337. 10.1186/s12877-019-1350-5 31791248 PMC6889660

[97] Atherton JJ , Sindone A, De Pasquale CG, Driscoll A, MacDonald PS, Hopper I, et al National Heart Foundation of Australia and Cardiac Society of Australia and New Zealand: guidelines for the prevention, detection, and management of heart failure in Australia 2018. Heart Lung Circ 2018;27:1123–208. 10.1016/j.hlc.2018.06.1042 30077227

[98] Schulberg SD , Ferry AV, Jin K, Marshall L, Neubeck L, Strachan FE, et al Cardiovascular risk communication strategies in primary prevention. A systematic review with narrative synthesis. J Adv Nurs 2022;78:3116–40. 10.1111/jan.15327 35719002 PMC9546276

[99] Bonner C , Batcup C, Cornell S, Fajardo MA, Hawkes AL, Trevena L, et al Interventions using heart age for cardiovascular disease risk communication: systematic review of psychological, behavioral, and clinical effects. JMIR Cardio 2021;5:e31056. 10.2196/31056 34738908 PMC8663444

[100] Allan GM , Nouri F, Korownyk C, Kolber MR, Vandermeer B, McCormack J. Agreement among cardiovascular disease risk calculators. Circulation 2013;127:1948–56. 10.1161/CIRCULATIONAHA.112.000412 23575355

[101] National Vascular Disease Prevention Alliance . Guidelines for the management of absolute cardiovascular disease risk. Sydney, Australia: Heart Foundation of Australia. ISBN: 978 – 0-9872830 –1-6. 2012.

[102] Navar AM , Stone NJ, Martin SS. What to say and how to say it: effective communication for cardiovascular disease prevention. Curr Opin Cardiol 2016;31:537–44. 10.1097/HCO.0000000000000322 27428113 PMC5045897

[103] Duval S , Van’t Hof JR, Steffen LM, Luepker RV. Estimation of cardiovascular risk from self-reported knowledge of risk factors: insights from the Minnesota Heart Survey. Clin Epidemiol 2020;12:41–9. 10.2147/CLEP.S219708 32021470 PMC6969684

[104] Prince SA , Cardilli L, Reed JL, Saunders TJ, Kite C, Douillette K, et al A comparison of self-reported and device measured sedentary behaviour in adults: a systematic review and meta-analysis. Int J Behav Nutr Phys Act 2020;17:31. 10.1186/s12966-020-00938-3 32131845 PMC7055033

[105] Cornelius T , Voils CI, Umland RC, Kronish IM. Validity of the self-reported domains of subjective extent of nonadherence (DOSE-nonadherence) scale in comparison with electronically monitored adherence to cardiovascular medications. Patient Prefer Adherence 2019;13:1677–84. 10.2147/PPA.S225460 31631982 PMC6781608

[106] Ogonowska-Slodownik A , Morgulec-Adamowicz N, Geigle PR, Kalbarczyk M, Kosmol A. Objective and self-reported assessment of physical activity of women over 60 years old. Ageing Int 2022;47:307–20. 10.1007/s12126-021-09423-z

[107] Schönfeld MS , Pfisterer-Heise S, Bergelt C. Self-reported health literacy and medication adherence in older adults: a systematic review. BMJ Open 2021;11:e056307. 10.1136/bmjopen-2021-056307 PMC8679075 34916329

[108] Doust JA , Bonner C, Bell KJL. Future directions in cardiovascular disease risk prediction. Aust J Gen Pract 2020;49:488–94. 10.31128/AJGP-02-20-5231 32738856

