# Supplementary material for: Cardiovascular disease risk communication and prevention: a meta-analysis
Source: Eur Heart J. 2024 Jan 19;45(12):998–1013. doi: 10.1093/eurheartj/ehae002 (PMC10972690; doi:10.1093/eurheartj/ehae002)
Supplement: ehae002_Supplementary_Data [file ehae002_supplementary_data.docx]

**Supplemental Material**

**Supplementary Box 1.** Search Strategy

**PubMed**

("Cardiovascular Diseases"[Mesh] OR "Cardiovascular disease"[tiab] OR "Cardiovascular diseases"[tiab] OR "Heart attacks"[tiab] OR "Heart attack"[tiab] OR “Heart disease”[tiab])

AND

("Health Communication"[Mesh] OR Communication[tiab] OR Communicate[tiab] OR Communicating[tiab] OR Message[tiab] OR Messages[tiab] OR "Risk information"[tiab] OR  "Providing information"[tiab] OR "General information"[tiab] OR Information[ti] OR Alerting[tiab] OR "Educational outreach"[tiab] OR "Health promotion"[tiab] OR Counseling[tiab] OR Counselling[tiab] OR Coaching[tiab] OR "Shared decision making"[tiab] OR Feedback[tiab] OR "Clinical decision support"[tiab] OR "Decision aid"[tiab] OR "Behavioral programs"[tiab] OR "Psychological impact"[tiab])

AND

("Risk Assessment"[Mesh] OR "Risk communication"[tiab] OR "Global risk"[tiab] OR "Risk perception"[tiab] OR "Risk appraisal"[tiab] OR "Risk assessment"[tiab] OR "Risk factor"[tiab] OR "Risk factors"[tiab] OR "Risk score"[tiab] OR "High threat message"[tiab] OR "Low threat message"[tiab] OR "Risk representations"[tiab] OR "Risk understanding"[tiab] OR "Risk comprehension"[tiab] OR "Cardiovascular risk"[tiab] OR "Increased risk"[tiab] OR "Risk information"[tiab] OR "Risk knowledge"[tiab])

AND

("Blood pressure"[ti] OR Hypertension[ti] OR "Cardiac events"[tiab] OR "CHD risk reduction"[tiab] OR Cholesterol[tiab] OR Prescriptions[tiab] OR "Heart age"[tiab] OR "Physical activity"[tiab] OR "Adherence score"[tiab] OR "Risk score"[tiab] OR "Mean score"[tiab] OR "GHQ-12 score"[tiab] OR "Risk perceptions"[tiab] OR "Perceived risk"[tiab] OR "Decisional conflict"[tiab])

AND

(Improving[tiab] OR Improvements[tiab] OR Improve[tiab] OR Reduction[tiab] OR Adherence[tiab] OR Increased[tiab] OR Higher[tiab] OR Accuracy[tiab] OR Influencing[tiab])

AND

((Randomized controlled trial[pt] OR controlled clinical trial[pt] OR randomized[tiab] OR randomised[tiab] OR "Experimental Study"[tiab] OR randomly[tiab] OR trial[tiab])

NOT

(Animals[Mesh] NOT (Animals[Mesh] AND Humans[Mesh]))

NOT

(“Case Reports”[pt] OR Editorial[pt] OR Letter[pt] OR Meta-Analysis[pt] OR “Observational Study”[pt] OR “Systematic Review”[pt] OR “Case Report”[ti] OR “Case series”[ti] OR Meta-Analysis[ti] OR “Meta Analysis”[ti] OR “Systematic Review”[ti]))

**Embase**

("cardiovascular disease"/exp/mj OR "Cardiovascular disease":ti,ab OR "Cardiovascular diseases":ti,ab OR "Heart attacks":ti,ab OR "Heart attack":ti,ab OR "Heart disease":ti,ab)

AND

("medical information"/exp/mj OR Communication:ti,ab OR Communicate:ti,ab OR Communicating:ti,ab OR Message:ti,ab OR Messages:ti,ab OR "Risk information":ti,ab OR "Providing information":ti,ab OR "General information":ti,ab OR Information:ti OR Alerting:ti,ab OR "Educational outreach":ti,ab OR "Health promotion":ti,ab OR Counseling:ti,ab OR Counselling:ti,ab OR Coaching:ti,ab OR "Shared decision making":ti,ab OR Feedback:ti,ab OR "Clinical decision support":ti,ab OR "Decision aid":ti,ab OR "Behavioral programs":ti,ab OR "Psychological impact":ti,ab)

AND

("Risk Assessment"/exp OR "Risk communication":ti,ab OR "Global risk":ti,ab OR "Risk perception":ti,ab OR "Risk appraisal":ti,ab OR "Risk assessment":ti,ab OR "Risk factor":ti,ab OR "Risk factors":ti,ab OR "Risk score":ti,ab OR "High threat message":ti,ab OR "Low threat message":ti,ab OR "Risk representations":ti,ab OR "Risk understanding":ti,ab OR "Risk comprehension":ti,ab OR "Cardiovascular risk":ti,ab OR "Increased risk":ti,ab OR "Risk information":ti,ab OR "Risk knowledge":ti,ab)

AND

("Blood pressure":ti OR Hypertension:ti OR "Cardiac events":ti,ab OR "CHD risk reduction":ti,ab OR Cholesterol:ti,ab OR Prescriptions:ti,ab OR "Heart age":ti,ab OR "Physical activity":ti,ab OR "Adherence score":ti,ab OR "Risk score":ti,ab OR "Mean score":ti,ab OR "GHQ-12 score":ti,ab OR "Risk perceptions":ti,ab OR "Perceived risk":ti,ab OR "Decisional conflict":ti,ab)

AND

(Improving:ti,ab OR Improvements:ti,ab OR Improve:ti,ab OR Reduction:ti,ab OR Adherence:ti,ab OR Increased:ti,ab OR Higher:ti,ab OR Accuracy:ti,ab OR Influencing:ti,ab)

AND

(random* OR factorial OR crossover OR placebo OR blind OR blinded OR assign OR assigned OR allocate OR allocated OR 'crossover procedure'/exp OR 'double-blind procedure'/exp OR 'randomized controlled trial'/exp OR 'single-blind procedure'/exp) NOT ('animal'/exp NOT ('animal'/exp AND 'human'/exp))

NOT

("Case Report":ti OR "Case series":ti OR Meta-Analysis:ti OR "Meta Analysis":ti OR "Systematic Review":ti)

**Supplementary Table 1.** List of excluded studies (n= 50)

**PsycINFO**

(exp "Cardiovascular Disorders"/ OR "Cardiovascular disease".ti,ab. OR "Cardiovascular diseases".ti,ab. OR "Heart attacks".ti,ab. OR "Heart attack".ti,ab. OR "Heart disease".ti,ab.)

AND

(exp "Communication"/ OR Communication.ti,ab. OR Communicate.ti,ab. OR Communicating.ti,ab. OR Message.ti,ab. OR Messages.ti,ab. OR "Risk information".ti,ab. OR "Providing information".ti,ab. OR "General information".ti,ab. OR Information.ti. OR Alerting.ti,ab. OR "Educational outreach".ti,ab. OR "Health promotion".ti,ab. OR Counseling.ti,ab. OR Counselling.ti,ab. OR Coaching.ti,ab. OR "Shared decision making".ti,ab. OR Feedback.ti,ab. OR "Clinical decision support".ti,ab. OR "Decision aid".ti,ab. OR "Behavioral programs".ti,ab. OR "Psychological impact".ti,ab.)

AND

(exp "Risk Assessment"/ OR "Risk communication".ti,ab. OR "Global risk".ti,ab. OR "Risk perception".ti,ab. OR "Risk appraisal".ti,ab. OR "Risk assessment".ti,ab. OR "Risk factor".ti,ab. OR "Risk factors".ti,ab. OR "Risk score".ti,ab. OR "High threat message".ti,ab. OR "Low threat message".ti,ab. OR "Risk representations".ti,ab. OR "Risk understanding".ti,ab. OR "Risk comprehension".ti,ab. OR "Cardiovascular risk".ti,ab. OR "Increased risk".ti,ab. OR "Risk information".ti,ab. OR "Risk knowledge".ti,ab.)

AND

("Blood pressure".ti. OR Hypertension.ti. OR "Cardiac events".ti,ab. OR "CHD risk reduction".ti,ab. OR Cholesterol.ti,ab. OR Prescriptions.ti,ab. OR "Heart age".ti,ab. OR "Physical activity".ti,ab. OR "Adherence score".ti,ab. OR "Risk score".ti,ab. OR "Mean score".ti,ab. OR "GHQ-12 score".ti,ab. OR "Risk perceptions".ti,ab. OR "Perceived risk".ti,ab. OR "Decisional conflict".ti,ab.)

AND

(Improving.ti,ab. OR Improvements.ti,ab. OR Improve.ti,ab. OR Reduction.ti,ab. OR Adherence.ti,ab. OR Increased.ti,ab. OR Higher.ti,ab. OR Accuracy.ti,ab. OR Influencing.ti,ab.)

AND

(control.tw. OR random.tw. OR exp treatment/)

NOT

("Case Reports".pt. OR Editorial.pt. OR Letter.pt. OR Meta-Analysis.pt. OR "Observational Study".pt. OR "Systematic Review".pt. OR "Case Report".ti. OR "Case series".ti. OR Meta-Analysis.ti. OR "Meta Analysis".ti. OR "Systematic Review".ti.)

|  | **Reference** | **Coded Reason for exclusion** | **Detailed justification** |
| --- | --- | --- | --- |
|  | Ahmadi M, Laumeier I, Ihl T, et al. A support programme for secondary prevention in patients with transient ischaemic attack and minor stroke (INSPiRE-TMS): an open-label, randomised controlled trial. *Lancet Neurol* 2020;19(1):49-60. doi: 10.1016/S1474-4422(19)30369-2 [published Online First: 2019/11/12] | Ineligible intervention | No outcome of interest: no obvious communication of cardiovascular risk |
|  | Alkhouli M, Kashem A, Homko CJ, et al. Patient-centered care of stage I and II hypertension in underserved communities. *Circulation: Cardiovascular Quality and Outcomes* 2011;4(6) | Ineligible intervention | No communication of cardiovascular risk |
|  | Bassett RL, Ginis KA. Risky business: the effects of an individualized health information intervention on health risk perceptions and leisure time physical activity among people with spinal cord injury. *Disabil Health J* 2011;4(3):165-76. doi: 10.1016/j.dhjo.2010.12.001 [published Online First: 2011/07/05] | Ineligible study design | Not a RCT |
|  | Buhse S, Kuniss N, Liethmann K, et al. An informed shared decision making programme for patients with type 2 diabetes in primary care: Cluster randomised controlled trial. *Diabetologia* 2017;60(1):S347. doi: 10.1007/s00125-017-4350-z | Conference abstract | included study |
|  | Carrington MJ, Schute F, Haluska B, et al. Preliminary results of the impress study: Reducing cardiovascular risk factors in a nurse-led primary intervention program for high risk participants. *European Heart Journal* 2012;33:1063. doi: 10.1093/eurheartj/ehs284 | Ineligible intervention | no obvious communication of cardiovascular risk |
|  | Charlson ME, Peterson JC, Boutin-Foster C, et al. Changing health behaviors to improve health outcomes after angioplasty: a randomized trial of net present value versus future value risk communication. *Health Educ Res* 2008;23(5):826-39. doi: 10.1093/her/cym068 | Ineligible intervention | net present  value’ economic theory, no outcomes of interest |
|  | Cioe PA, Merrill JE, Gordon REF, et al. Personalized feedback improves cardiovascular risk perception and physical activity levels in persons with HIV: results of a pilot randomized clinical trial. *AIDS Care* 2021;33(6):786-94. doi: 10.1080/09540121.2021.1874271 | Others  Duplicate | Included study duplicate |
|  | Cobos A, Vilaseca J, Asenjo C, et al. Cost effectiveness of a clinical decision support system based on the recommendations of the European Society of Cardiology and other societies for the management of hypercholesterolemia: Report of a cluster-randomized trial. *Disease Management and Health Outcomes* 2005;13(6):421-32. doi: 10.2165/00115677-200513060-00007 | Ineligible intervention | no obvious communication of cardiovascular risk |
|  | Coylewright M, Dick S, Zmolek B, et al. PCI Choice Decision Aid for Stable Coronary Artery Disease: A Randomized Trial. *Circ Cardiovasc Qual Outcomes* 2016;9(6):767-76. doi: 10.1161/circoutcomes.116.002641 | Ineligible intervention | Not about CV risk communication |
|  | Cykert S, Keyserling T, DeWalt D, et al. A controlled trial of dissemination and implementation of a cardiovascular risk reduction strategy in small primary care practices. *Health Services Research* 2020;55(SUPPL 1):80-81. doi: 10.1111/1475-6773.13441 | Conference abstract | No enough data for ROB |
|  | Edelman D, Wray L, Nieuwsma J, et al. Randomized trial of group problem solving for cardiovascular risk reduction. *Journal of General Internal Medicine* 2019;34(2):S340. doi: 10.1007/11606.1525-1497 | Ineligible intervention | Not about CV risk communication |
|  | Evans CD, Eurich DT, Taylor JG, et al. The Collaborative Cardiovascular Risk Reduction in Primary Care (CCARP) study. *Pharmacotherapy* 2010;30(8):766-75. doi: 10.1592/phco.30.8.766 [published Online First: 2010/07/27] | Ineligible intervention | Not cleat if CV risk was communicated to patients |
|  | Fornasini M, Baldeon ME, Zevallos JC, et al. Impact of Training Primary Care Physicians in Behavioral Counseling to Reduce Cardiovascular Disease Risk Factors in Quito, Ecuador. *Faseb J* 2017;31(1) | Ineligible intervention | CV risk was not communicated to patients |
|  | Gryn S, Har B, Dresser G. Evaluation of the effect of cardiovascular risk assessment on treatment compliance in hypertension. *Journal of Clinical Hypertension* 2012;14 | Conference abstract | No enough data for ROB |
|  | Gulayin PE, Lozada A, Beratarrechea A, et al. An Educational Intervention to Improve Statin Use: Cluster RCT at the Primary Care Level in Argentina. *Am J Prev Med* 2019;57(1):95-105. doi: 10.1016/j.amepre.2019.02.018 | Ineligible intervention | CV risk was not communicated to patients |
|  | Harmsen CG, Jarbøl DE, Nexøe J, et al. Impact of effectiveness information format on patient choice of therapy and satisfaction with decisions about chronic disease medication: the "Influence of intervention Methodologies on Patient Choice of Therapy (IMPACT)" cluster-randomised trial in general practice. *BMC Health Serv Res* 2013;13:76. doi: 10.1186/1472-6963-13-76 | Others  (Study protocol) | Protocol of an excluded study |
|  | Harmsen CG, Kristiansen IS, Larsen PV, et al. Communicating risk using absolute risk reduction or prolongation of life formats: cluster-randomised trial in general practice. *Br J Gen Pract* 2014;64(621):e199-207. doi: 10.3399/bjgp14X677824 | Ineligible intervention | Not about communicating CV risk |
|  | Hetlevik I, Holmen J, Krüger O, et al. Implementing clinical guidelines in the treatment of hypertension in general practice. *Blood Press* 1998;7(5-6):270-6. doi: 10.1080/080370598437114 | Ineligible intervention | Not about communicating CV risk |
|  | Jaspers NEM, Visseren FLJ, Van Der Graaf Y, et al. Effects of personalized therapy-effect predictions on statin treatment decisions by patients and physicians: A three-armed, blinded, randomized controlled trial. *European Heart Journal* 2019;40:255. doi: 10.1093/eurheartj/ehz747.0253 | Conference abstract | Not enough data for ROB |
|  | John H, Hale ED, Treharne GJ, et al. A randomized controlled trial of a cognitive behavioural patient education intervention versus a traditional information leaflet to address the cardiovascular aspects of rheumatoid disease. *Rheumatology (United Kingdom)* 2012;51:iii77. doi: 10.1093/rheumatology/kes110 | Conference abstract | Not enough data for ROB |
|  | Jorstad HT, von Birgelen C, Alings AM, et al. Effect of a nurse-coordinated prevention programme on cardiovascular risk after an acute coronary syndrome: main results of the RESPONSE randomised trial. *Heart* 2013;99(19):1421-30. doi: 10.1136/heartjnl-2013-303989 | Ineligible intervention | CV risk was not communicated |
|  | Katz DA, Graber M, Lounsbury P, et al. Multiple Risk Factor Counseling to Promote Heart-healthy Lifestyles in the Chest Pain Observation Unit: Pilot Randomized Controlled Trial. *Acad Emerg Med* 2017;24(8):968-82. doi: 10.1111/acem.13231 | Ineligible intervention | though they have a CV risk assessment it is not reported that it was communicated to the patient |
|  | Kavita, Thakur JS, Vijayvergiya R, et al. Task shifting of cardiovascular risk assessment and communication by nurses for primary and secondary prevention of cardiovascular diseases in a tertiary health care setting of Northern India. *BMC Health Serv Res* 2020;20(1):10. doi: 10.1186/s12913-019-4864-9 | Ineligible study design | Quasi-experimental and the trial for secondary prevention had no outcomes of interest |
|  | Lalonde L, O'Connor AM, Duguay P, et al. Evaluation of a decision aid and a personal risk profile in community pharmacy for patients considering options to improve cardiovascular health: The OPTIONS pilot study. *International Journal of Pharmacy Practice* 2006;14(1):51-62. doi: 10.1211/ijpp.14.1.0007 | Ineligible population | Too small sample size |
|  | Langham J, Tucker H, Sloan D, et al. Secondary prevention of cardiovascular disease: a randomised trial of training in information management, evidence-based medicine, both or neither: the PIER trial. *Br J Gen Pract* 2002;52(483):818-24. | Ineligible intervention | Not about CV risk communication |
|  | Lee TJ, Cameron LD, Wunsche B, et al. A randomized trial of computer-based communications using imagery and text information to alter representations of heart disease risk and motivate protective behaviour. *Br J Health Psychol* 2011;16(Pt 1):72-91. doi: 10.1348/135910710X511709 [published Online First: 2011/01/14] | Ineligible population | Mean age 21.5 years |
|  | Mack MC. The effects of health coaching on cardiovascular risk reduction among adult patients with history of hypertension. *Dissertation Abstracts International Section A: Humanities and Social Sciences* 2020;81(10-A):No Pagination Specified. | Ineligible intervention | no use of risk tools, no outcome of interest |
|  | Peiris D, Usherwood T, Panaretto K, et al. O105 Effect Of A Multi-Faceted Quality Improvement Intervention To Improve Cardiovascular Disease Risk Identification And Management In Australian Primary Health Care: The Torpedo Cluster-Randomised Trial. *Global Heart* 2014;9(1):e28. doi: 10.1016/j.gheart.2014.03.1317 | Conference abstract only | insufficient data for RoB |
|  | Plante TB, Juraschek SP. 10 year ASCVD RISK AND INCIDENT ASCVD IN THE SYSTOLIC BLOOD PRESSURE INTERVENTION TRIAL (SPRINT). *Journal of General Internal Medicine* 2017;32(2):S89-S90. | Conference abstract only | insufficient data for RoB |
|  | Reid C, McNeil JJ, Williams F, et al. Cardiovascular risk reduction: a randomized trial of two health promotion strategies for lowering risk in a community with low socioeconomic status. *J Cardiovasc Risk* 1995;2(2):155-63. [published Online First: 1995/04/01] | Ineligible population | participants aged 18-65 and >120 aged under 30, results not presented separately |
|  | Saffi MA, Polanczyk CA, Rabelo-Silva ER. Lifestyle interventions reduce cardiovascular risk in patients with coronary artery disease: a randomized clinical trial. *Eur J Cardiovasc Nurs* 2014;13(5):436-43. doi: 10.1177/1474515113505396 | Ineligible intervention | No indication that risk score was communicated to patients as part of the intervention (even if all other outcomes are relevant) |
|  | Salazar A, Vilchez-Barboza V, Saez K, et al. Effectiveness of nursing counseling for patients with cardiovascular risk factors: Controlled clinical trial with a 24 months post-intervention follow-up. *European Heart Journal* 2019;40:636. doi: 10.1093/eurheartj/ehz748.0002 | Conference abstract only | insufficient data for RoB |
|  | Schwalm J, McCready T, Lamelas P, et al. The Heart Outcomes Prevention Evaluation 4 Canada: A Pilot Study of a Community-Based, Multi-Faceted Intervention, Led by Non-Physicians to Reduce Cardiovascular Risk in People with Hypertension. *Canadian Journal of Cardiology* 2019;35(10):S22-S23. doi: 10.1016/j.cjca.2019.07.398 | Conference abstract only | insufficient data for RoB |
|  | Schwalm JD, McCready T, Lopez-Jaramillo P, et al. A community-based comprehensive intervention to reduce cardiovascular risk in hypertension (HOPE 4): a cluster-randomised controlled trial. *Lancet* 2019;394(10205):1231-42. doi: 10.1016/S0140-6736(19)31949-X [published Online First: 2019/09/07] | Ineligible intervention | no evidence that the risk score was communicated to patients; this is a CVD RF reduction trial where multicomponent interventon was decided before risk determined |
|  | Selvaraj FJ, Mohamed M, Omar K, et al. The impact of a disease management program (COACH) on the attainment of better cardiovascular risk control in dyslipidaemic patients at primary care centres (The DISSEMINATE Study): a randomised controlled trial. *BMC Fam Pract* 2012;13:97. doi: 10.1186/1471-2296-13-97 | Ineligible intervention | No evidence that risk was communicated to patients- implementation trial |
|  | Shah S, Brown T, Lee JY, et al. Individualized Risk Communication and Lay Outreach for the Primary Prevention of Cardiovascular Disease in Community Health Centers: Preliminary Results of a Randomized Controlled Trial. *Journal of General Internal Medicine* 2014;29:S126-S26. | Conference abstract only | insufficient data for RoB |
|  | Sheridan SL, Draeger LB, Pignone M, et al. Patients' Choices for Lifestyle Change Versus Medication Use to Reduce Elevated Cvd Risk. *Journal of General Internal Medicine* 2012;27:S268-S68. | Conference abstract only | insufficient data for RoB |
|  | Sheridan SL, Keyserling TC, Draeger LB. A Randomized Trial of a Web-Based Versus Counselor-Based Intervention to Reduce Chd Risk. *Journal of General Internal Medicine* 2013;28:S13-S13. | Conference abstract only | insufficient data for RoB |
|  | Silarova B, Lucas J, Butterworth AS, et al. Information and Risk Modification Trial (INFORM): design of a randomised controlled trial of communicating different types of information about coronary heart disease risk, alongside lifestyle advice, to achieve change in health-related behaviour. *BMC Public Health* 2015;15:868. doi: 10.1186/s12889-015-2192-5 [published Online First: 2015/09/09] | Others  (Study protocol) | Included study |
|  | Siren R, Eriksson JG, Vanhanen H. Observed changes in cardiovascular risk factors among high-risk middle-aged men who received lifestyle counselling: A 5-year follow-up. *Scandinavian Journal of Primary Health Care* 2016;34(4):336-42. doi: http://dx.doi.org/10.1080/02813432.2016.1248649 | Ineligible study design | No intervention- Observational study- Ineligible study design |
|  | Staten LK, Gregory-Mercado KY, Ranger-Moore J, et al. Provider counseling, health education, and community health workers: the Arizona WISEWOMAN project. *J Womens Health (Larchmt)* 2004;13(5):547-56. doi: 10.1089/1540999041281133 | Ineligible intervention | No mention of risk scores as part of intervention |
|  | Stoddard AM, Palombo R, Troped PJ, et al. Cardiovascular disease risk reduction: the Massachusetts WISEWOMAN project. *J Womens Health (Larchmt)* 2004;13(5):539-46. doi: 10.1089/1540999041281106 | Ineligible intervention | No mention of risk scores as part of intervention |
|  | Svendsen K, Telle-Hansen V, Garstad KW, et al. Changes in lifestyle and risk factor levels after identification of and alerting to high cardiovascular risk: A randomized controlled trial of healthy subjects visiting Norwegian pharmaciesGrant from Mills DA and regular research funding from the University of Oslo. *European Journal of Preventive Cardiology* 2016;23:S26-S27. doi: 10.1177/2047487316668118 | Conference abstract only | insufficient data for RoB |
|  | Taveira TH, Dooley AG, Cohen LB, et al. Pharmacist-led group medical appointments for the management of type 2 diabetes with comorbid depression in older adults. *Ann Pharmacother* 2011;45(11):1346-55. doi: 10.1345/aph.1Q212 | Ineligible intervention | No evidence of risk score communication as part of the intervention |
|  | Vilchez Barboza V, Klijn TP, Salazar Molina A, et al. Effectiveness of personalized face-to-face and telephone nursing counseling interventions for cardiovascular risk factors: a controlled clinical trial. *Rev Lat Am Enfermagem* 2016;24:e2747. doi: 10.1590/1518-8345.0626.2747 [published Online First: 2016/08/11] | Ineligible intervention | No evidence of risk score communication as part of the intervention |
|  | Wang C, Sen A, Ruffin MT, et al. Family history assessment: Impact on disease risk perceptions. *Am J Prev Med* 2012;43(4):392-98. doi: 10.1016/j.amepre.2012.06.013 | Ineligible intervention | accuracy of CVD risk perception using a tool but on several illnesses, not based on a risk score but generic question (weak, strong familial risk) |
|  | Willaing I, Ladelund S, Jørgensen T, et al. Nutritional counselling in primary health care: a randomized comparison of an intervention by general practitioner or dietician. *Eur J Cardiovasc Prev Rehabil* 2004;11(6):513-20. doi: 10.1097/01.hjr.0000152244.58950.5f | Ineligible intervention | No evidence of Danish risk score communication as part of the intervention |
|  | Wong MCS, Wang HHX, Kwan MWM, et al. The effectiveness of Dietary Approaches to Stop Hypertension (DASH) counselling on estimated 10-year cardiovascular risk among patients with newly diagnosed grade 1 hypertension: A randomised clinical trial. *Int J Cardiol* 2016;224:79-87. doi: 10.1016/j.ijcard.2016.08.334 | Ineligible intervention | No evidence of risk score communication as part of the intervention |
|  | Zhang H, Jiang Y, Nguyen HD, et al. The effect of a smartphone-based coronary heart disease prevention (SBCHDP) programme on awareness and knowledge of CHD, stress, and cardiac-related lifestyle behaviours among the working population in Singapore: a pilot randomised controlled trial. *Health Qual Life Outcomes* 2017;15(1):49. doi: 10.1186/s12955-017-0623-y | Ineligible population | >50% were aged under 30 years and results not presented separately |
|  | Zheng XJ, Yu HB, Qiu XCH, et al. The effects of a nurse-led lifestyle intervention program on cardiovascular risk, self-efficacy and health promoting behaviours among patients with metabolic syndrome: Randomized controlled trial. *Int J Nurs Stud* 2020;109:103638. doi: ARTN 103638 | Ineligible intervention | No evidence that risk score was communicated |
|  | Aycock DM, Clark PC, Hayat MJ, et al. Stroke Counseling Intervention for Young Adult African Americans: A Randomized Controlled Trial. Nurs Res 2023;72(2):83-92. doi: 10.1097/nnr.0000000000000633 [published Online First: 20221209] | Ineligible population | The study included participations < 30 years old |
|  | Baldeón ME, Fornasini M, Flores N, et al. Impact of training primary care physicians in behavioral counseling to reduce cardiovascular disease risk factors in Ecuador. Revista Panamericana de Salud Publica/Pan American Journal of Public Health 2018;42:e139. doi: 10.26633/RPSP.2018.139 [published Online First: 20180924] | Ineligible intervention | No evidence of risk score communication as part of the intervention |
|  | Balducci S, Haxhi J, Sacchetti M, et al. Relationships of Changes in Physical Activity and Sedentary Behavior With Changes in Physical Fitness and Cardiometabolic Risk Profile in Individuals With Type 2 Diabetes: The Italian Diabetes and Exercise Study 2 (IDES_2). Diabetes Care 2022;45(1):213-21. doi: 10.2337/dc21-1505 | Ineligible intervention | No evidence of risk score communication as part of the intervention |
|  | Bengtsson A, Norberg M, Ng N, et al. Pictorial information about subclinical atherosclerosis reduces the CVD risk: Results from the VIPVIZA RCT. European Journal of Preventive Cardiology 2021;28(SUPPL 1):i228. doi: 10.1093/eurjpc/zwab061.248 | Ineligible intervention | No evidence of risk score communication as part of the intervention |
|  | Castro M, Vijay A, Govind N, et al. evalUation of imPact of a novel immersive technology in patient engagement of LIFe simple 7 to prevent risk of hearT disease: UPLIFT clinical trial. Postgraduate Medicine 2023;135(sup2):10-11. doi: 10.1080/00325481.2023.2187148 | Conference abstract only | insufficient data for RoB and outcome data |
|  | Cioe PA, Merrill JE, Gordon RE, et al. Personalized feedback improves cardiovascular risk perception and physical activity levels in persons with hiv: Results of a pilot randomized clinical trial. AIDS Care 2021;33(6):No Pagination Specified. doi: https://dx.doi.org/10.1080/09540121.2021.1874271 [published Online First: 20210123] | Others  Duplicate | Study already included |
|  | collaborators SIt. Secondary prevention with a structured semi-interactive stroke prevention package in INDIA (SPRINT INDIA): a multicentre, randomised controlled trial. Lancet Glob Health 2023;11(3):e425-e35. doi: 10.1016/s2214-109x(22)00544-7 | Ineligible intervention | No evidence of risk score communication as part of the intervention |
|  | Gidlow CJ, Ellis NJ, Cowap L, et al. Cardiovascular disease risk communication in nhs health checks using qrisk® 2 and jbs3 risk calculators: The rico qualitative and quantitative study. Health Technology Assessment 2021;25(50):VII-102. doi: 10.3310/hta25500 | Ineligible study design | Qualitative study design with process evaluation |
|  | Halmesvaara O, Vornanen M, Kääriäinen H, et al. Psychosocial Effects of Receiving Genome-Wide Polygenic Risk Information Concerning Type 2 Diabetes and Coronary Heart Disease: A Randomized Controlled Trial. Front Genet 2022;13:881349. doi: 10.3389/fgene.2022.881349 [published Online First: 20220530] | Ineligible population | The study included participations < 30 years old |
|  | Jeemon P, Harikrishnan S, Ganapathi S, et al. Efficacy of a family-based cardiovascular risk reduction intervention in individuals with a family history of premature coronary heart disease in India (PROLIFIC): an open-label, single-centre, cluster randomised controlled trial. The Lancet Global Health 2021;9(10):e1442-e50. doi: 10.1016/S2214-109X(21)00319-3 | Ineligible intervention | No evidence of risk score communication as part of the intervention |
|  | Kask-Flight L, Durak K, Suija K, et al. Reduction of cardiovascular risk factors among young men with hypertension using an interactive decision aid: cluster-randomized control trial. BMC Cardiovasc Disord 2021;21(1):543. doi: 10.1186/s12872-021-02339-1 [published Online First: 20211116] | Others  Duplicate | Study already included |
|  | Kidanu AW, Shi R, Cruz-Cano R, et al. Health Information on Waterpipe Lounge Menus to Educate Young Adults: Pilot Study Findings. Health Educ Behav 2022;49(4):618-28. doi: 10.1177/10901981211020990 | Ineligible intervention | Health education for young adults |
|  | Klimis H, Thiagalingam A, McIntyre D, et al. Text messages for primary prevention of cardiovascular disease: The TextMe2 randomized clinical trial. Am Heart J 2021;242:33-44. doi: 10.1016/j.ahj.2021.08.009 | Ineligible intervention | No evidence of risk score communication as part of the intervention |
|  | Kunneman M, Branda M, Hargraves I, et al. Shared Decision Making for Atrial Fibrillation: An Overview of Seven Studies Within an Encounter -Randomized Trial. Circulation 2022;146 doi: 10.1161/circ.146.suppl_1.9623 | Others  Study protocol | Ongoing study |
|  | Olomu A, Tikaria R, Kelly-Blake K, et al. Type 2 diabetes patient activation and mHealth interventions decreased cardiovascular disease risk. Am J Manag Care 2022;28(11):e392-e98. doi: 10.37765/ajmc.2022.89263 | Ineligible intervention | No evidence of risk score communication as part of the intervention |
|  | Taksler GB, Hu B, DeGrandis F, Jr., et al. Effect of Individualized Preventive Care Recommendations vs Usual Care on Patient Interest and Use of Recommendations: A Pilot Randomized Clinical Trial. JAMA Netw Open 2021;4(11):e2131455. doi: 10.1001/jamanetworkopen.2021.31455 [published Online First: 20211101] | Others  Duplicate | Study already included |
|  | Verma KP, Marwick TH, Duarte C, et al. Use of coronary computed tomography or polygenic risk scores to prompt action to reduce coronary artery disease risk: the CAPAR-CAD trial. Am Heart J 2022;248:97-107. doi: 10.1016/j.ahj.2022.02.007 | Others | Ongoing study |
|  | Waters EA, Maki J, Liu Y, et al. Risk ladder, table, or bulleted list? Identifying formats that effectively communicate personalized risk and risk reduction information for multiple diseases. Medical Decision Making 2021;41(1):74-88. doi: https://dx.doi.org/10.1177/0272989X20968070 | Ineligible intervention | Multiple risk scores for different conditions were communicated |

**CV: cardiovascular, CVD: cardiovascular risk, RoB: risk of bias, RCT: randomised controlled trial**

**Supplementary Table 2.** Characteristics of trial interventions

| *Study ID* | Type of comparator^[[1]](#footnote-2)^ | | | Intervention characteristics^[[2]](#footnote-3)^ | | | | | | | | | | | | | | | | | | |
| --- | --- | --- | --- | --- | --- | --- | --- | --- | --- | --- | --- | --- | --- | --- | --- | --- | --- | --- | --- | --- | --- | --- |
|  | Usual care | Extended usual care^[[3]](#footnote-4)^ | Active comparator^[[4]](#footnote-5)^ | Single component | Multifaceted | Delivery | | Level of implementation | | | Intervention components/CV risk communication tools | | | | Intervention function^[[5]](#footnote-6)^ | | | | | | **Who provided the intervention?** | How often CV risk information was provided (or intervention containing CV risk)? |
|  |  |  |  |  |  | F2F^[[6]](#footnote-7)^ | Remote^[[7]](#footnote-8)^ | Patient | Clinician | System/organisation | Patient materials^[[8]](#footnote-9)^ | Clinician tools^[[9]](#footnote-10)^ | Electronic^[[10]](#footnote-11)^ | Verbal disclosure^[[11]](#footnote-12)^ | Education | Persuasion | Training | Enablement | Environmental re-structuring | Modelling |  |  |
| Family Heart Study Group, 1994 | ✔ |  |  |  | 3 | ✔ |  | ✔ | ✔ |  | P/Bk |  |  | ✔ | ✔^p^ |  | ✔^c^ |  |  | ✔^p^ | Nurses | Every 2,3,4, or 6 months (according to degree of risk) |
| Adarkwah, 2019 |  |  | DA | ✔ |  | ✔ |  | ✔ | ✔ |  |  |  | DA | ✔ | ✔^p^ |  |  |  | ✔^c^ |  | GPs | 1 |
| Avis, 1989 |  |  | RS | ✔ |  | ✔ |  | ✔ |  |  | RS |  |  |  | ✔^p^ |  |  |  |  |  | Tech | 1 |
| Ayres, 2012 | ✔ |  | RS | ✔ |  |  | W | ✔ |  |  |  |  | RS |  | ✔^p^ |  |  | ✔^p^ |  |  | Researchers | 1 |
| Benner, 2008 | ✔ |  |  |  | 3 | ✔ |  | ✔ | ✔ |  | RS |  | RS | ✔ | ✔^p^ |  | ✔^c^ |  | ✔^c^ |  | Physicians | 1 |
| Bonner, 2015 |  |  | RS | ✔ |  |  | W | ✔ |  |  |  |  | RS |  | ✔^p^ |  |  |  |  |  | Researchers | 1 |
| Bonner, 2022 |  |  | RS | ✔ |  |  | W | ✔ |  |  |  |  | RS |  | ✔^p^ |  |  |  |  |  | Researchers | 1 |
| Broadbent, 2013 | ✔ |  |  | ✔ |  | ✔ |  | ✔ | ✔ |  | RS |  | DSS |  |  |  | ✔^c^ | ✔^p^ | ✔^c^ |  | Nurses | 2 * 30 minutes |
| Brotons, 2021 | ✔ |  |  | ✔ |  | ✔ |  | ✔ | ✔ |  | RS |  |  |  | ✔^p^ |  | ✔^p^ |  |  |  | Nurses | 4 per year |
| Bucher, 2010 |  | BK |  |  | 2 | ✔ |  |  | ✔ |  |  | BK/RS |  | ✔ | ✔^c^ |  |  |  | ✔^c^ |  | Physicians | 1 |
| Buhse, 2018 |  | GL |  |  | 5 | ✔ |  | ✔ | ✔ |  | DS | DA |  |  | ✔^p^ | ✔^p^ | ✔^c^ | ✔^p^ |  |  | GPs | 1*90 minutes |
| Buhse, 2015 |  | BK |  |  | 5 | ✔ |  | ✔ | ✔ |  | DS | DA |  |  | ✔^p^ | ✔^p^ | ✔^c^ | ✔^p^ |  |  | GPs | 1*90 minutes |
| Christensen, 2004 | ✔ |  |  | ✔ |  | ✔ |  | ✔ |  |  | F |  |  | ✔ | ✔^p^ |  |  | ✔^p^ |  |  | GPs | 1*45-min |
| Cioe, 2021 | ✔ |  |  | ✔ |  | ✔ |  | ✔ |  |  |  |  |  | ✔ | ✔^p^ | ✔^p^ |  | ✔^p^ |  |  | Nurses | 2*45-min |
| Edelman, 2006 |  | RS |  |  | 5 | ✔ |  | ✔ |  |  | DS |  |  | ✔ | ✔^p^ | ✔^p^ | ✔^p^ |  |  |  | Health coach/ Physician assistant | 2 (1 & 5 months) |
| Escortell-Mayor, 2020 |  |  | VD/Tr |  | 3 | ✔ |  | ✔ | ✔ |  | P/RS |  | RS | ✔ | ✔^p^ |  | ✔^c^ | ✔^p^ | ✔^c^ |  | Family doctors & nurses | 1*5-10 min |
| Fernandez, 2009 | ✔ |  |  |  | 5 | ✔ |  | ✔ | ✔ |  | BK/ Leaf |  |  | ✔ | ✔^c^ ✔^p^ | ✔^p^ |  | ✔^p^ |  | ✔^p^ | GP /research assistant | 1 |
| Grover, 2007 | ✔ |  |  | ✔ |  | ✔ |  | ✔ |  |  | RS | RS |  | ✔ | ✔^p^ | ✔^p^ |  |  | ✔^c^ | ✔^p^ | Physicians | 5 (Baseline, 3,6,9,12 months) |
| Hanlon, 1995 | ✔ |  |  | ✔ |  | ✔ |  | ✔ |  |  |  |  |  | ✔ | ✔^p^ | ✔^p^ |  |  |  |  | NR | 3 (baseline, 5 & 12 months) |
| Hess, 2016 | ✔ |  |  | ✔ |  | ✔ |  | ✔ | ✔ |  |  | DA |  |  | ✔^p^ |  | ✔^c^ | ✔^p^ | ✔^c^ |  | Clinicians | 1 |
| Hess, 2012 | ✔ |  |  | ✔ |  | ✔ |  | ✔ | ✔ |  |  | DA |  |  | ✔^p^ |  | ✔^c^ | ✔^p^ | ✔^c^ |  | Clinicians | 1 |
| Jacobson, 2006 |  | R |  | ✔ |  | ✔ |  |  | ✔ |  |  | RS |  | ✔ | ✔^p^ |  |  |  | ✔^c^ |  | Clinicians | 1 |
| Jaspers, 2021 | ✔ |  |  |  | 3 |  | T+L | ✔ |  |  | Leaf |  | Vid^p^ |  | ✔^p^ | ✔^p^ |  |  |  | ✔^p^ | GPs | 1 |
| Kask-Flight, 2021 | ✔ |  |  | ✔ |  | ✔ |  |  | ✔ |  |  |  | DA | ✔ |  |  | ✔^c^ |  |  |  | Family doctors | 1 |
| Koelewijn-van Loon, 2009 |  | Tr |  |  | 2 | ✔ |  |  | ✔ | ✔ | DA | DA |  |  | ✔^p^ | ✔^p^ | ✔^c^ |  |  | ✔^p^ | Nurses | 2*15-20 min |
| Krones, 2008 |  | Sr |  | ✔ |  | ✔ |  |  | ✔ |  |  |  | DA | ✔ |  |  | ✔^c^ | ✔^c^ |  |  | Family doctors | 1 |
| Kunneman, 2020 | ✔ |  |  | ✔ |  | ✔ |  |  | ✔ |  | Leaf | DA |  | ✔ | ✔^p^ |  | ✔^c^ |  |  |  | Clinicians | 1 |
| Lopez-Gonzalez, 2015 | ✔ |  |  | ✔ |  | ✔ |  |  | ✔ |  |  |  |  | ✔ | ✔^p^ |  | ✔^c^ |  |  |  | Researchers & clinical assistants | 1 |
| Lovibond, 1986 |  | B |  |  | 5 | ✔ |  | ✔ |  |  |  |  | Vid^p^ | ✔ | ✔^p^ | ✔^p^ |  | ✔^p^ |  |  | Psychologist | Multiple (over 6months) |
| Lowensteyn, 1998 | ✔ |  |  | ✔ |  | ✔ |  |  | ✔ |  | RS | RS |  | ✔ | ✔^p^ |  |  |  | ✔^c^ |  | Physicians | 1 or 2 |
| Mann, 2010 |  | R |  | ✔ |  | ✔ |  |  | ✔ |  |  | DA |  | ✔ | ✔^p^ |  |  | ✔^p^ |  |  | Clinicians | 1*6-min |
| Maron, 2008 |  | R/Ed |  | ✔ |  | ✔ |  | ✔ |  |  | RS |  |  | ✔ | ✔^p^ |  |  | ✔^p^ |  |  | Nurses | Multiple-15 min |
| Mitchell, 2005 | ✔ |  |  | ✔ |  | ✔ |  |  | ✔ |  |  | RS |  |  |  |  |  | ✔^c^ |  |  | GPs | 1 |
| Montgomery, 2003 |  | R |  | ✔ |  | ✔ |  | ✔ |  |  | RS |  | Vid^p^ | ✔ | ✔^p^ | ✔^p^ |  | ✔^p^ |  |  | Researchers | 1 |
| Montgomery, 2000 | ✔ |  |  | ✔ |  | ✔ |  |  | ✔ |  | RS |  | DSS | ✔ | ✔^p^ |  | ✔^c^ |  |  |  | GPs and nurses | 1 |
| Mortsiefer, 2015 |  | GL/Leaf |  |  | 5 | ✔ |  |  | ✔ |  | Leaf | Bk |  | ✔ | ✔^p^ ✔^c^ | ✔^c^ |  | ✔^p^ ✔^c^ |  | ✔^c^ | GPs | Multiple |
| Muscat 2021 | ✔ |  |  | ✔ |  |  | W | ✔ |  |  |  |  | RS |  | ✔^p^ |  |  |  |  |  | Researchers | 1 |
| Nieuwkerk, 2012 | ✔ |  |  | ✔ |  | ✔ |  | ✔ |  |  | RS |  |  | ✔ | ✔^p^ |  |  | ✔^p^ |  |  | Nurses | 4 (Baseline, 3,9, & 18) |
| Nolan, 2011 |  |  | RS/B |  | 3 |  | TH | ✔ |  |  | RS |  |  |  | ✔^p^ |  |  | ✔^p^ |  |  | Family doctors | 1 |
| Oddone, 2018 |  |  | RS |  | 2 | ✔ | T | ✔ |  |  |  |  | RS | ✔ | ✔^p^ | ✔^p^ | ✔^c^ | ✔^p^ |  |  | Health coach | 1 |
| Peiris, 2015 | ✔ |  |  |  | 4 | ✔ |  |  | ✔ | ✔ |  |  | DSS/ RS | ✔ | ✔^c^ |  | ✔^c^ | ✔^c^ | ✔^c^ |  | GPs | 1 |
| Perestelo-Perez, 2016 | ✔ |  |  | ✔ |  | ✔ |  | ✔ | ✔ |  | DA | DA |  | ✔ | ✔^p^ |  |  | ✔^p^ |  |  | Physicians | 1 |
| Persell, 2015 | ✔ |  |  |  | 2 |  | L/T | ✔ |  |  | RS |  |  | T | ✔^p^ | ✔^p^ | ✔^p^ | ✔^p^ |  |  | Care managers | 1 |
| Persell, 2013 | ✔ |  |  | ✔ |  |  | L | ✔ |  |  | RS |  |  |  | ✔^p^ | ✔^p^ |  |  |  |  | Researchers | 1 |
| Powers, 2011 |  | Edu/R |  | ✔ |  | ✔ |  | ✔ |  |  | RS | RS |  |  | ✔^p^ |  |  | ✔^p^ |  |  | Researchers | 1 |
| Redfern, 2020 | ✔ |  |  |  | 6 |  | W | ✔ |  |  | RS/R/DS |  |  |  | ✔^p^ | ✔^p^ | ✔^p^ | ✔^p^ |  |  | Researchers | Multiple |
| Sarfo, 2023 | ✔ |  |  |  | 4 |  | W | ✔ |  |  |  |  | RS +vid |  | ✔^p^ |  |  |  |  |  | Researchers | NR |
| Sheridan, 2011 | ✔ |  |  | ✔ |  |  | W | ✔ |  |  | RS |  | DA/M |  | ✔^p^ | ✔^p^ |  | ✔^p^ |  |  | Researchers | NR |
| Sheridan, 2006 |  | RL |  | ✔ |  |  | W | ✔ |  |  | RS |  | DA/M |  | ✔^p^ | ✔^p^ |  | ✔^p^ |  |  | Researchers | NR |
| Silarova, 2019 | ✔ |  |  | ✔ |  |  | W | ✔ |  |  |  |  | RS /M |  | ✔^p^ | ✔^p^ |  | ✔^p^ |  |  | Researchers | 3 |
| Soureti, 2010 |  |  | HA | ✔ |  |  | W | ✔ |  |  |  |  | RS |  | ✔^p^ |  |  | ✔^p^ |  |  | Researchers | 1 |
| Soureti, 2011 | ✔ | B |  | ✔ |  |  | W | ✔ |  |  |  |  | RS |  | ✔^p^ |  |  | ✔^p^ |  |  | Researchers | 1 |
| Svendsen, 2020 |  |  | VD | ✔ |  | ✔ |  | ✔ | ✔ |  | RS | RS |  | ✔ | ✔^p^ | ✔^p^ | ✔^c^ | ✔^c^✔^p^ |  | ✔^p^ | Pharmacy-staff | 1 |
| Svendsen, 2018 | ✔ |  |  | ✔ |  | ✔ |  | ✔ |  |  | L |  |  | ✔ | ✔^p^ | ✔^p^ |  |  |  |  | Pharmacy-staff | 1 |
| Taksler, 2021 | ✔ |  |  | ✔ |  | ✔ |  |  | ✔ |  |  | RS | RS | ✔ | ✔^p^ | ✔^p^ |  | ✔^c^ | ✔^c^ |  | Physicians | 1 |
| Tinsel, 2013 | ✔ |  |  | ✔ |  | ✔ |  |  | ✔ |  |  |  |  | ✔ |  | ✔^p^ | ✔^c^ | ✔^c^ |  |  | GPs | NR |
| Turnbull, 2006 | ?? |  |  |  | ✔ | ✔ | ✔ | ✔ |  |  | ✔ |  |  |  | ✔ |  |  |  | ✔ |  | GPs/dieticians | Multiple (non-standardised #) |
| van Steenkiste, 2007 |  | R |  |  | 3 | ✔ |  | ✔ | ✔ |  | BK/DS | DSS/Bk |  |  | ✔^p^ | ✔^p^ | ✔^c^ | ✔^p^ |  |  | GPs | 2 (Baseline, 1-2 weeks) |
| Viigimaa, 2022 | ✔ |  |  | ✔ |  | ✔ | W | ✔ |  |  |  |  | RS |  | ✔^p^ |  |  |  |  |  | GPs | 3 (baseline, 3months, 12 months) |
| Weymiller, 2007 |  | P |  | ✔ |  | ✔ | W | ✔ | ✔ |  | DA | DA |  |  | ✔^p^ | ✔^p^ |  | ✔^p^ |  |  | Endocrinologists | 1 |
| Wister, 2007 | ✔ |  |  |  | 2 | ✔ | TH | ✔ |  |  | RS/R |  |  |  | ✔^p^ | ✔^p^ | ✔^c^ | ✔^p^ |  |  | Family doctor/clinical lifestyle counsellors | 2 per year* 30-60 minutes |
| Zullig, 2014 |  | R |  |  | 3 | ✔ | W | ✔ |  |  |  |  | RS/R/M |  | ✔^p^ | ✔^p^ |  | ✔^p^ |  |  | Self-administered web risk calculator | 1 |

T: Telephone counselling/follow-up, W: web-based/online-counselling, TH: Telehealth counselling, L: Letters/mailed information, DSS: Decision-support system, P: pamphlets, BK: Booklet, DA: Decision aid, GPs: General Practitioners, Tech: technicians, RS: Risk appraisal/score tool visualising patient’s CVD risk, Tr: Training, DS: documentation sheet, GL: guideline, F: Feedback, R: general resources, VD: verbal disclosure, NR: Not reported, Vid: Video, Leaf: leaflet/report, Sr: Seminar, B: basic behaviour intervention, Ed: Education, e-R: electronic general resources, M: Messages, RL: List of risks, HA: Heart age

**Supplementary Table 3.** Characteristics of included studies

| Study ID | Location | Design | | | | Participants | | | | Setting | | | |
| --- | --- | --- | --- | --- | --- | --- | --- | --- | --- | --- | --- | --- | --- |
|  |  | RCT | C-RCT | No. of arms | No of clusters  (if C-RCT) | Number  Patients* (clinicians) | Without CVD (primary prevention) | With CVD (secondary prevention) | Age range/mean (SD) in years | Community | Primary care | Secondary | Tertiary care |
| Family Heart Study Group, 1994 | UK |  | ✔ | 2 | 26 | 12472 | ✔ |  | 40-59 |  | ✔ |  |  |
| Adarkwah, 2019 | Germany | ✔ |  | 2 |  | 304 (33) | ✔ |  | 30–80 |  | ✔ |  |  |
| Avis, 1989 | USA | ✔ |  | 5 |  | 732 | ✔ |  | 25-65** | ✔ |  |  |  |
| Ayres, 2012 | UK | ✔ |  | 4 |  | 292 | ✔ |  | 52.9 (9.8)*** | ✔ |  |  |  |
| Benner, 2008 | Multi-EU |  | ✔ | 2 | 100 | 1103 | ✔ |  | 56.8 (5.1) |  | ✔ |  |  |
| Bonner, 2015 | Australia | ✔ |  | 6 |  | 570 | ✔ |  | 54 (6) | ✔ |  |  |  |
| Bonner, 2022 | Australia | ✔ |  | 6 |  | 859 | ✔ |  | 45-74 | ✔ |  |  |  |
| Broadbent, 2013 | NZ | ✔ |  | 2 |  | 130 |  | ✔ | 58.6 (11.80) |  |  |  | ✔ |
| Brotons, 2021 | Spain | ✔ |  | 2 |  | 464 | ✔ |  | 61 (8) |  | ✔ |  |  |
| Bucher, 2010 | Switzerland |  | ✔ | 2 |  | 4097 (165) | ✔ | ✔ | 39-51 |  |  | ✔ | ✔ |
| Buhse, 2018 | Germany | ✔ |  | 2 |  | 307 | ✔ |  | 58.7 (7.9) *** |  |  | ✔ |  |
| Buhse, 2015 | Germany | ✔ |  | 2 |  | 154 | ✔ |  | 61.7 (6.5) *** |  |  | ✔ |  |
| Christensen, 2004 | Denmark | ✔ |  | 3 |  | 2000 | ✔ |  | 30–49 |  | ✔ |  |  |
| Cioe, 2021 | USA | ✔ |  | 2 |  | 40 | ✔ |  | 51.5 (7.4) |  |  | ✔ |  |
| Edelman, 2006 | USA | ✔ |  | 2 |  | 154 | ✔ |  | 53.4 (4.8) *** | ✔ |  |  |  |
| Escortell-Mayor, 2020 | Spain |  | ✔ | 2 | 22 | 411 |  | ✔ | 55.3 (6.7) |  | ✔ |  |  |
| Fernandez, 2009 | Australia | ✔ |  | 2 |  | 51 |  | ✔ | 57 (8.78) |  |  |  | ✔ |
| Grover, 2007 | Canada | ✔ |  | 2 |  | 3053 |  | ✔ | 56.3 (7.9) |  | ✔ |  |  |
| Hanlon, 1995 | UK | ✔ |  | 2 |  | 1600 | ✔ |  | 20-65** | ✔ |  |  |  |
| Hess, 2016 | USA | ✔ |  | 2 |  | 913 (361) |  | ✔ | 50.3 (14.5) |  |  |  | ✔ |
| Hess, 2012 | USA | ✔ |  | 2 |  | 208 | ✔ |  | 54.9 (12) *** |  |  |  | ✔ |
| Jacobson, 2006 | USA | ✔ |  | 2 |  | 368 | ✔ |  | 58 (9) |  |  |  | ✔ |
| Jaspers, 2021 | Netherlands | ✔ |  | 3 |  | 303 |  | ✔ | 58-71 |  | ✔ |  |  |
| Kask-Flight, 2021 | Estonia |  | ✔ | 2 | 20 | 77 | ✔ |  | 36.98 (7.93) |  | ✔ |  |  |
| Koelewijn-van Loon, 2009 | Netherlands |  | ✔ | 2 | 25 | 615 | ✔ |  | 58 (10) *** |  | ✔ |  |  |
| Krones, 2008 | Germany |  | ✔ | 2 | 14 | 1132 (91) | ✔ |  | 58.6 (12.5) *** |  | ✔ |  |  |
| Kunneman, 2020 | USA | ✔ |  | 2 |  | 922 (244) |  | ✔ | 71 (11) |  | ✔ | ✔ | ✔ |
| Lopez-Gonzalez, 2015 | Spain | ✔ |  | 3 |  | 3153 |  | ✔ | 46.1 (7.1) | ✔ |  |  |  |
| Lovibond, 1986 | Australia | ✔ |  | 3 |  | 75 | ✔ |  | 30-60 | ✔ |  |  |  |
| Lowensteyn, 1998 | Canada | ✔ |  | 2 |  | 958 (253) | ✔ |  | 50.5 (10.8) |  | ✔ |  |  |
| Mann, 2010 | USA | ✔ |  | 2 |  | 150 (46) |  | ✔ | 58 (11) *** |  | ✔ |  |  |
| Maron, 2008 | USA | ✔ |  | 2 |  | 133 |  | ✔ | 46.8 (8.6) | ✔ |  |  |  |
| Mitchell, 2005 | UK |  | ✔ | 3 | 54 | 1755 |  | ✔ | 65-79 |  | ✔ |  |  |
| Montgomery, 2003 | UK | ✔ |  | 4 |  | 217 | ✔ |  | 32-80 |  | ✔ |  |  |
| Montgomery, 2000 | UK |  | ✔ | 3 | 27 | 614 | ✔ | ✔ | 60-79 |  | ✔ |  |  |
| Mortsiefer, 2015 | Germany |  | ✔ | 2 | 89 | 3355 (89) | ✔ |  | 40-75 |  | ✔ |  |  |
| Muscat 2021 | Australia | ✔ |  | 3 |  | 1318 | ✔ |  | 40 to 50 | ✔ |  |  |  |
| Nieuwkerk, 2012 | Netherlands | ✔ |  | 2 |  | 201 |  | ✔ | 49.2 (1.3) *** |  |  | ✔ |  |
| Nolan, 2011 | Canada | ✔ |  | 2 |  | 680 |  | ✔ | 35-74 | ✔ | ✔ |  |  |
| Oddone, 2018 | USA | ✔ |  | 2 |  | 417 | ✔ |  | 55.8 (12.2) |  | ✔ |  |  |
| Peiris, 2015 | Australia |  | ✔ | 2 | 61 | 38,725 | ✔ | ✔ | 61 (NR) |  | ✔ |  |  |
| Perestelo-Perez, 2016 | Spain |  | ✔ | 2 | 14 | 168 (29) | ✔ |  | 59.6 (12.3) |  | ✔ |  |  |
| Persell, 2015 | USA | ✔ |  | 2 |  | 646 | ✔ |  | 59.7 (9) |  | ✔ |  |  |
| Persell, 2013 | USA |  | ✔ | 2 | 29 | 435 (29) | ✔ |  | 40-79 |  | ✔ |  |  |
| Powers, 2011 | UK | ✔ |  | 2 |  | 89 |  | ✔ | 67 (8) |  | ✔ |  |  |
| Redfern, 2020 | Australia | ✔ |  | 2 |  | 934 |  | ✔ | 67.6 (8.1) |  | ✔ |  |  |
| Sarfo, 2023 | Nigeria | ✔ |  | 2 |  | 100 | ✔ |  | 59.5 (12.5) | ✔ |  |  |  |
| Sheridan, 2011 | USA | ✔ |  | 2 |  | 165 | ✔ |  | 40-79 |  |  | ✔ |  |
| Sheridan, 2006 | USA | ✔ |  | 2 |  | 75 | ✔ |  | 53 (9) |  |  | ✔ |  |
| Silarova, 2019 | UK | ✔ |  | 4 |  | 956 | ✔ |  | 56.7 (8.8) |  | ✔ |  |  |
| Soureti, 2010 | Netherlands | ✔ |  | 2 |  | 413 | ✔ |  | 30-60 | ✔ |  |  |  |
| Soureti, 2011 | Netherlands | ✔ |  | 4 |  | 781 | ✔ |  | 30-60 | ✔ |  |  |  |
| Svendsen, 2020 | Norway |  | ✔ | 2 | 48 | 508 (48) | ✔ |  | 60.5 (12.9) |  | ✔ |  |  |
| Svendsen, 2018 | Norway | ✔ |  | 3 |  | 582 | ✔ |  | 56.5 (14.6) |  | ✔ |  |  |
| Taksler, 2021 | USA | ✔ |  | 2 |  | 73 | ✔ |  | 45-70 |  | ✔ |  |  |
| Tinsel, 2013 | Germany |  | ✔ | 2 | 37 | 1120 | ✔ | ✔ | 65 (12.4) *** |  | ✔ |  |  |
| Turnbull, 2006 | Australia |  | ✔ | 2 | 67 | 371 | ✔ |  | 60.5 (9.5) |  | ✔ |  |  |
| van Steenkiste, 2007 | Netherlands |  | ✔ | 2 | 34 | 490 | ✔ |  | 40–75 |  | ✔ |  |  |
| Viigimaa, 2022 | Estonia | ✔ |  | 2 |  | 1018 | ✔ |  | 51.2 (9.8) |  | ✔ |  |  |
| Weymiller, 2007 | USA |  | ✔ | 2 | 21 | 98 | ✔ |  | 66 (8) *** |  |  | ✔ |  |
| Wister, 2007 | Canada | ✔ |  | 2 |  | 611 | ✔ | ✔ | 45–64 | ✔ |  |  |  |
| Zullig, 2014 | USA | ✔ |  | 2 |  | 96 |  | ✔ | 63.1 (12.2) |  | ✔ |  |  |

*Number of participants randomised

**Mean age is more than 30 years

***Mean age reported for the control groupCVD: cardiovascular disease, no.: number, RCT: randomised controlled trial, C-RCT: cluster randomised controlled trial, SD: standard deviation, USA: United States America, UK: United Kingdom

**
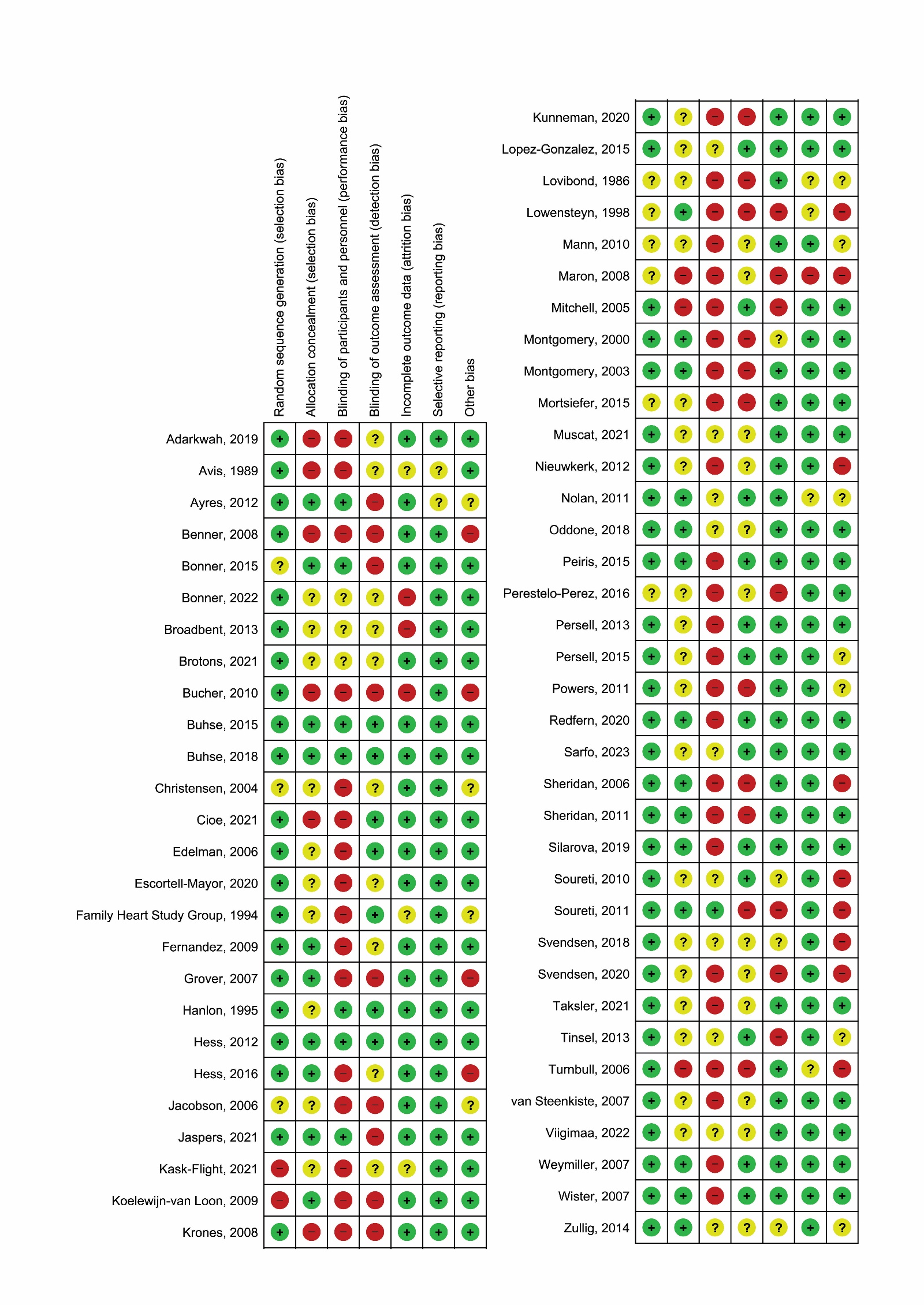
**

**Supplementary Figure 1.** Risk of bias summary

**Box 2.** Funnel plots comparisons


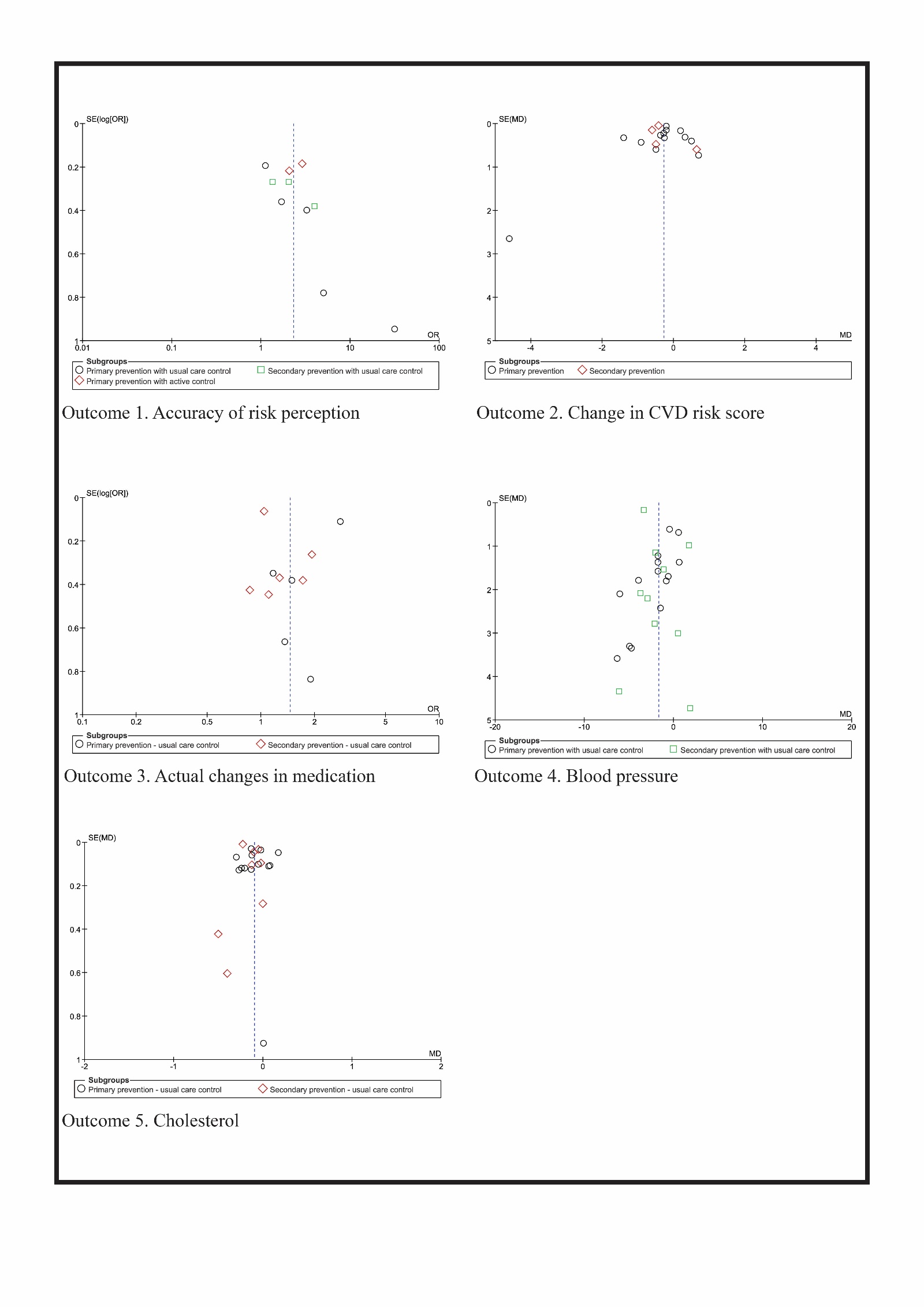


**
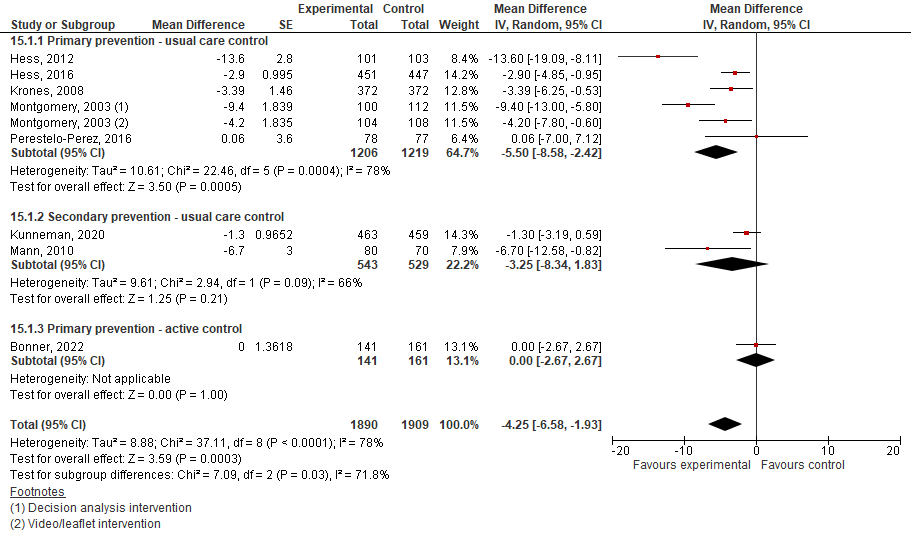
**

**Supplementary Figure 2. Impact of CVD risk communication on decisional conflict (n=9 studies)**

**
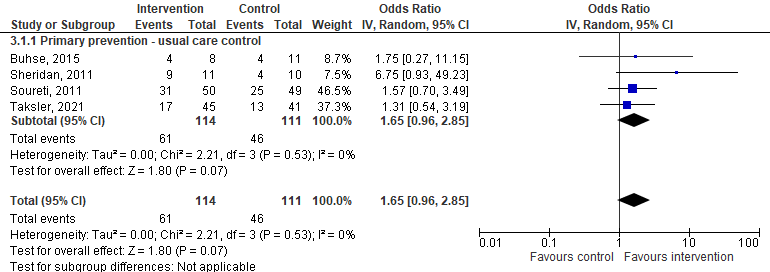
**

**Supplementary Figure 3. Panel A:** Intention to stop smoking (n=4 studies)

**
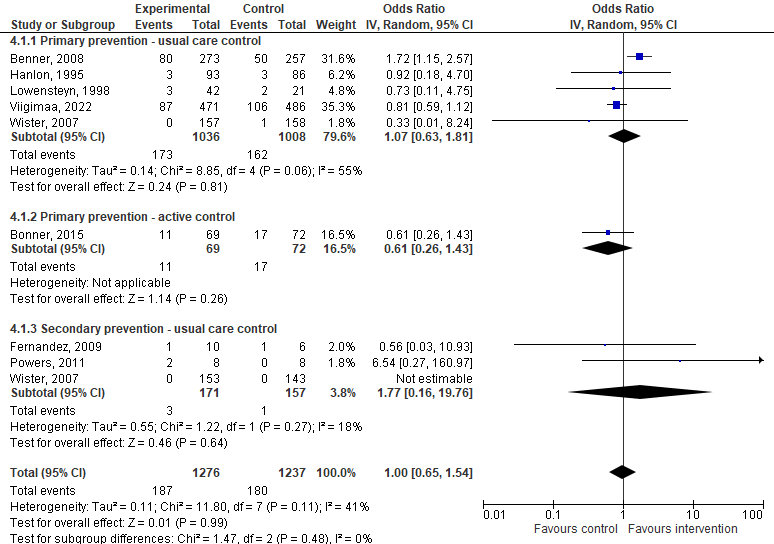
**

**Supplementary Figure 3. Panel B:** Self-reported smoking cessation at follow-up (n=8 studies)

**Supplementary Figure 3.** Impact of CVD risk communication on intention to cease smoking (Panel A) and self-report of smoking cessation (Panel B)

**
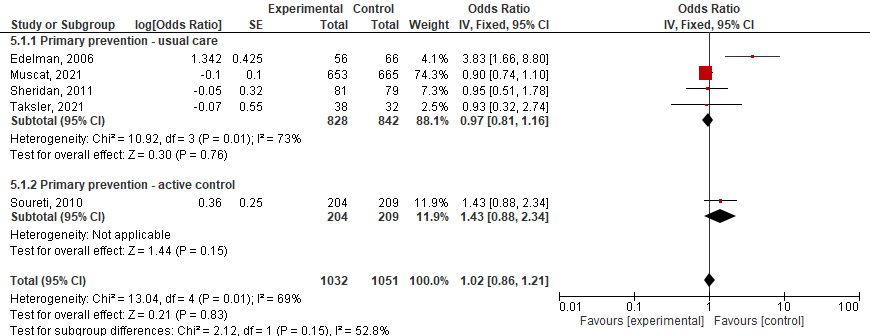
**

**Supplementary Figure 4. Panel A:** Physical activity intentions (n=5 studies)

**
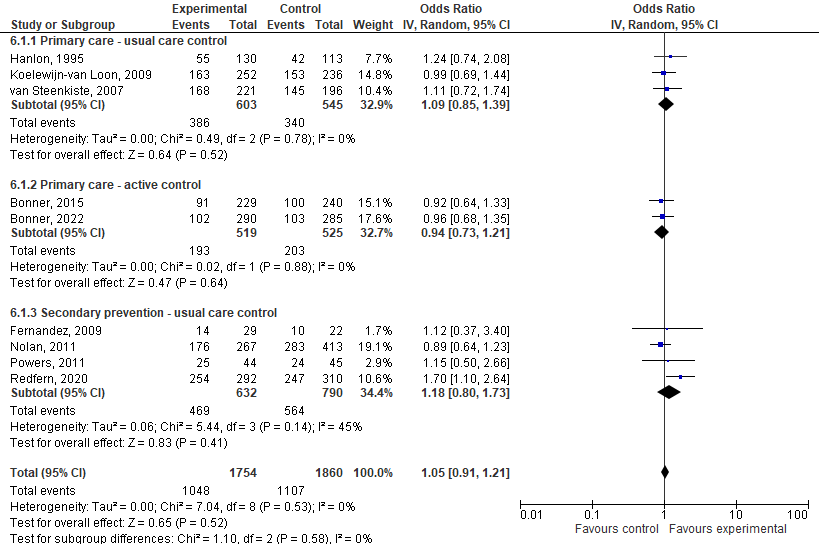
**

**Supplementary Figure 4. Panel B:** Self-reported physical activity change at follow-up (n= 9 studies)

**Supplementary Figure 4.** Impact of CVD risk communication on Physical activity intention (panel A) and self-report of physical activity change (panel B)

**
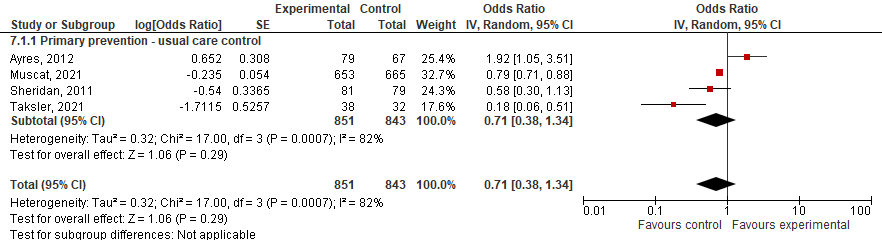
**

**Supplementary Figure 5. Panel A:** Intention to modify diet (n=4 studies)

**
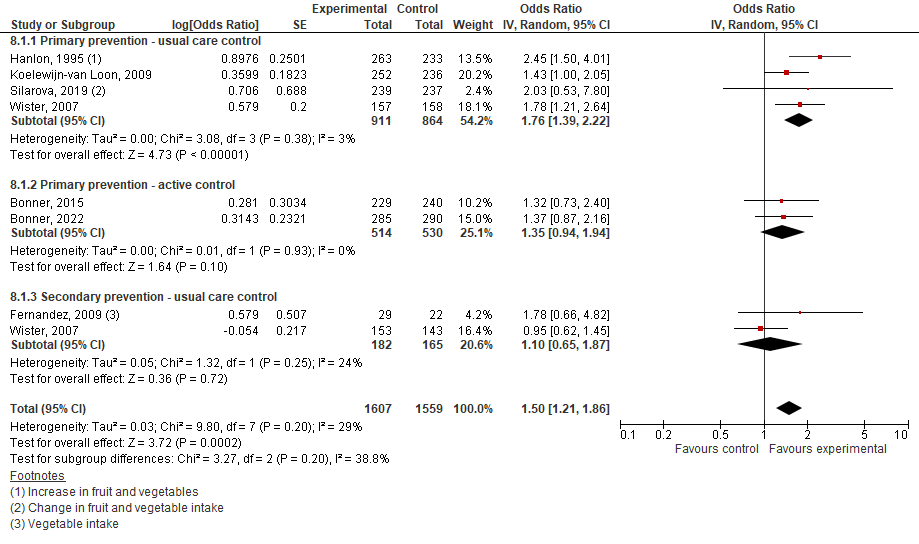
**

**Supplementary Figure 5. Panel B:** Self-report of actual dietary change at follow-up (7 studies)

**Supplementary Figure 5.** Impact of CVD risk communication on Intention to modify diet (panel A) and self-report of actual dietary change (panel B).

**
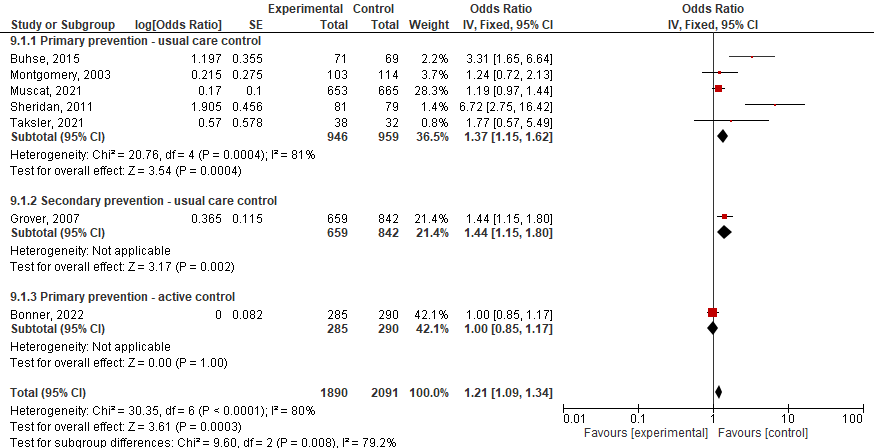
**

**Supplementary Figure 6. Panel A:** Intention to initiate/change medication (n=7 studies)


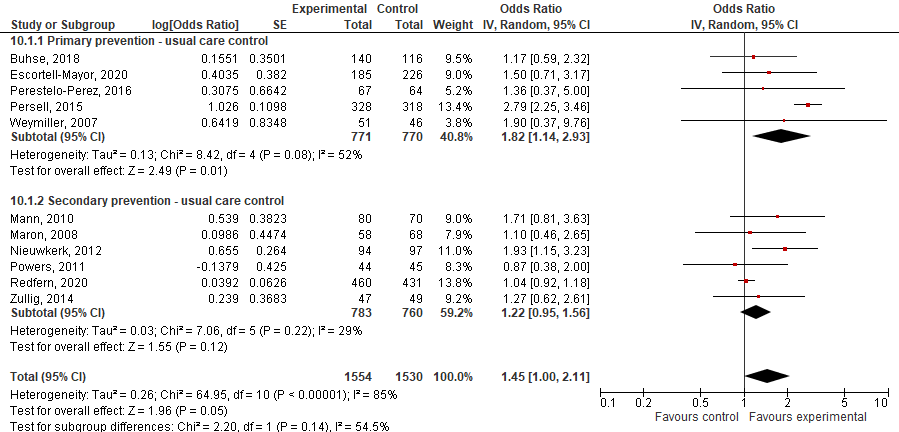


**Supplementary Figure 6. Panel B:** Self-reported initiation/change in medication at follow-up (n=11 studies)

**Supplementary Figure 6.** Impact of CVD risk communication on Intention to initiate/change medication (panel A) and patient reported medication change (panel B)

**
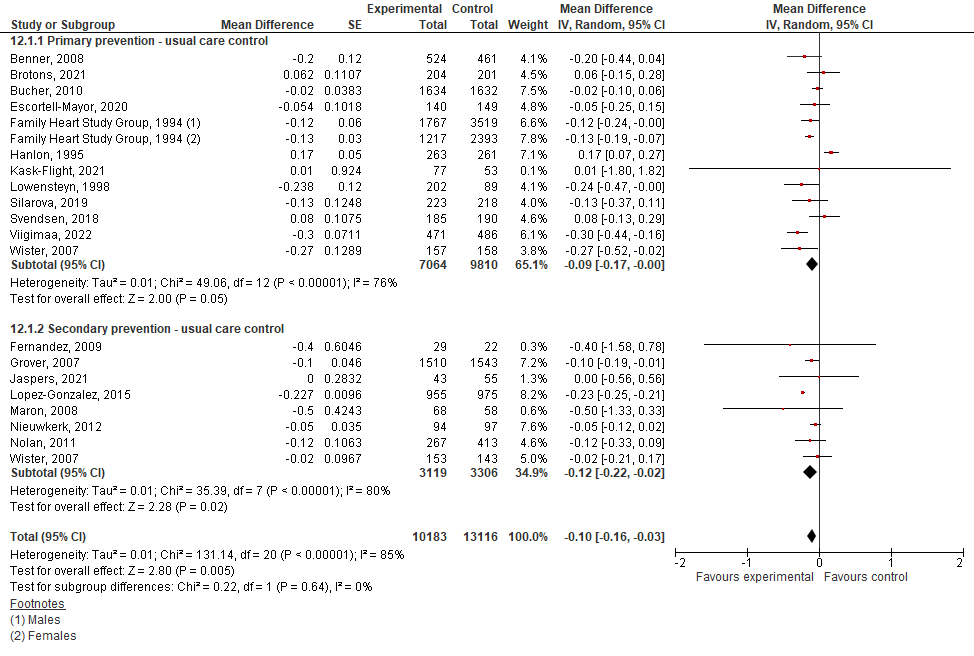
**

**Supplementary Figure 7.** Impact of CVD risk communication on follow-up blood cholesterol (n=20 studies)

**
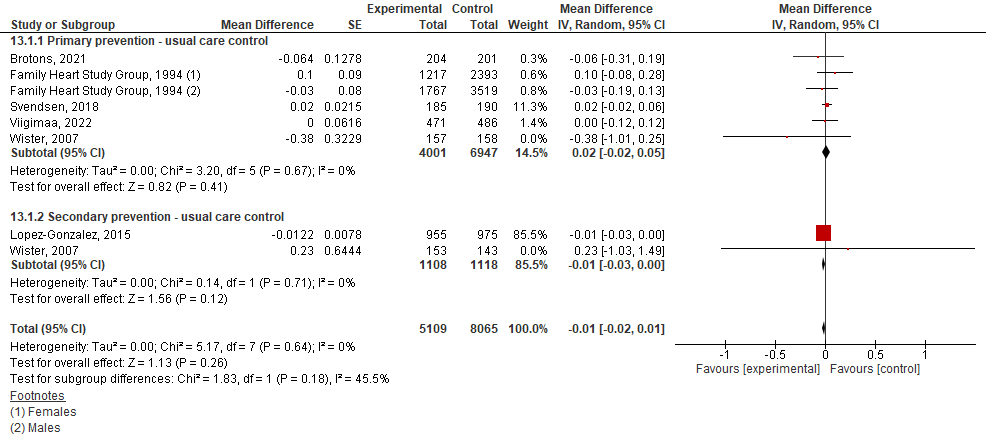
**

**Supplementary Figure 8.** Impact of CVD risk communication on follow-up blood glucose (n=7 studies)

**
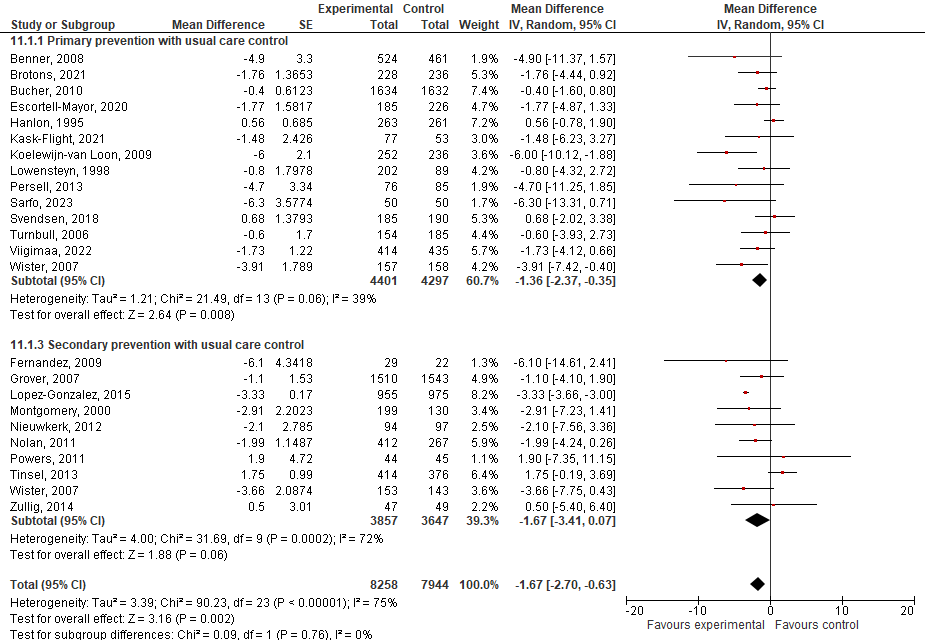
**

**Supplementary Figure 9.** Impact of CVD risk communication on follow-up blood pressure (n=23 studies)

**
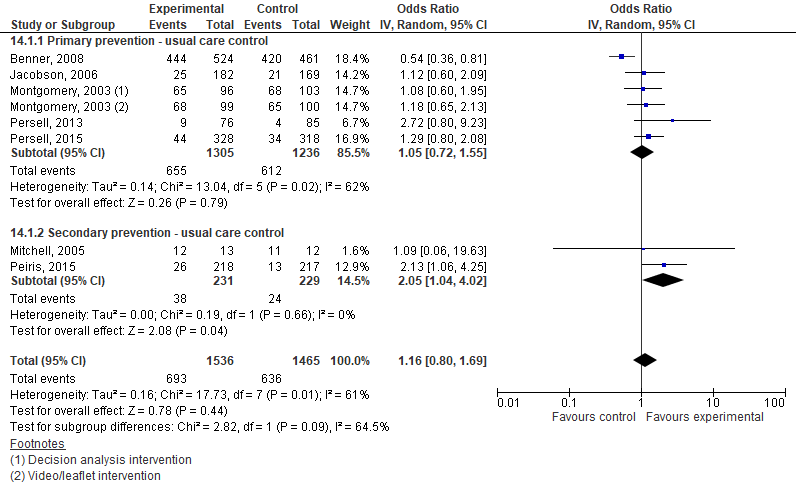
**

**Supplementary Figure 10.** Impact of CVD risk communication on clinician’s prescribing (n= 7 studies)

**Supplementary Table 4.** Findings from studies non meta-analysable

|  |  |  | **Primary prevention** | | **Secondary prevention** | |
| --- | --- | --- | --- | --- | --- | --- |
|  | **Timepoint &**  **reference** | **Number of participants** | **Intervention** | **control** | **Intervention** | **control** |
| **Accuracy of risk perception** | | | | | | |
| Change in accuracy of objective risk | Immediate &  3months  Avis 1989 |  | 40% accurate only after feedback (overall for all risk levels) | N/R |  |  |
| Realistic expectation of risk | Immediate & 6+ months  Buhse 2015 | 143 | **Immediate Mean realistic risk expectation score (SD)**  **4.51 (1.61)**  **6-months mean realistic risk score** 1.41 | Immediate Mean realistic risk expectation score (SD)  0.85 (0.98)  **Immediate Diff 3.67 (95%CI 3.23, 4.11) p<0.001**  **6-months mean realistic risk** 0.90  **6-month Diff 0.51**  **(95% CI 0.09 to 0.93); p=0.018** |  |  |
| Accuracy of risk perception with or without medication | 3 months  Perestelo-Perez, 2016 | 159 | Accurate risk perception with statins  48.1%  **Accurate risk perception without statins**  **42.0%** | Accurate risk perception with statins  29.5% Diff p=0.08  **Accurate risk perception without statins**  **26.9% Diff p=0.013** |  |  |
| Absolute individual and comparative risk perception | 3 months  Silarova 2019 | 861 | Mean change in 2 arms with CVD risk disclosure  Absolute perceived risk  -13.71 (55.9)  -4.99 (57.9)  Comparative risk  -0.13 (0.68)  -0.09 (0.72) | Mean change in 2 arms without CVD risk disclosure  Absolute perceived risk  -8.75 (98.5)  -3.67 (55.2)  p=0.565  Comparative risk  -0.20 (0.75)  -0.14 (0.66)  p=0.674 |  |  |
| Individual and comparative risk perception | Immediate & 4 weeks  Soureti 2011 | 781 | Between group diff  -Immediate individual  -Comparative  **4 weeks**  **Comparative with Heart Age message**  **p=0.001** | p=0.88  p=0.93  4 weeks  Comparative without Heart Age message  p=0.34 |  |  |
| Accuracy of risk perception with or without medication | Immediate, 2-3m, 6-9m  Mann 2010 | 150 |  |  | Accurate risk perception with statins 43.0%  Accurate risk perception without statins  44.0% | Accurate risk perception with statins  35.0% Diff p=NS  Accurate risk perception without statins  29.0% Diff p=NS |
| Adequate knowledge of risk | 6-9 months  Buhse 2018 | 279 | 44.9% | 2.8%  P <0.001 |  |  |
| Accurate perception of high individual or comparative risk | immediate  Soureti 2010 | 413 | Individual mean (SD)  9.7 (6.0)  Comparative mean (SD)  10.2 (5.8) | Individual mean (SD)  14.4 (8.0) Diff p=0.477  Comparative mean (SD)  12.1 (8.6) Diff p=0.993 |  |  |
| **Change in CVD risk score** | | | | | | |
| Change in CVD risk scores | 3 months  Kask-Flight, 2021 | 130 | 10-year risk of stroke or heart attack  7.83 (0.97)  Lifetime risk of CVD  61.35 (1.6) | 10-year risk of stroke or heart attack  7.89 (1.51)  Mean diff −0.57  p=0.429  Lifetime risk of CVD  61.7 (2)  Mean diff +0.30  p=0.773 |  |  |
| 5-yr and 10-yr change in CVD Risk score | 6 &12 months  Lovibond, 1986 | 75 | 6-m Mean reduction 0.076  12-m Mean reduction 0.071 | 6-m Mean reduction 0.061 control1  0.04 control 2 Diff NS  12-m Mean reduction 0.059 control1  0.035 control 2 Diff NS |  |  |
| 10-yr mean Framingham risk score | 12 months  Maron, 2008 | 133 |  |  | **Mean score 4.5**  **1-yr decrease -1.33 or 22.6%** | **Mean score 4.8**  **1-year increase +0.20**  **Or 4.3%**  **p=0.013** |
| Change in perceived risk awareness score | 3 months  Cioe 2021 | 40 | Increase +0.38 | Reduction -0.30  P=0.19 |  |  |
| Change in CVD risk score | 2 months  Svendsen 2018 | 543 | **Alert-advice Reduction −0.36 (95% CI:−0.62, −0.09)**  Advice only Reduction −0.71 (95% CI:−0.99, −0.44) | **Reduction −0.76 (95% CI:−1.02, −0.50)**  **Diff p=0.03**  Diff p=0.8 |  |  |
| Change in perceived mean risk | 3 months  Powers 2011 | 89 |  |  | Personalised (SD)  39.5 (4.5) | Standard (SD)  38.2 (4.5)  Diff 3.6 (-8.1, 15.4)  p=0.54 |
| Change in 10-yr CVD risk mean score | 3 months  Zullig 2014 | 96 |  |  | 0.2 (0.0) | 0.3 (0.0)  P=0.81 |
| change in absolute 8-yr CVD risk score | 12 months  Lowensteyn 1998 | 291 | **mean (SD)**  **-1.8 (4.7)** | **Mean (SD)**  **-0.3 (5.3)**  **Diff -1.426 p<0.01** |  |  |
| % reduction in risk score | 12 months  FHS 1994 | 12,472 | **Vs.external control**  **Men**  **Women**  **Vs.t internal control**  **Men**  **Women** | **% (95% CI)**  **16.01 (10.9, 21.1)**  **15.7 (7.4, 23.3)**  **17.6 (14.0, 21.2)**  **13.2 (7.3, 18.6)** |  |  |
| Change in mean absolute risk score | 12 months  Montgomery 2000 | 531 |  |  | DA+chart +0.7  Chart only -0.5 | Usual care: 0.8  P= N/R but NS |
| **Emotional Impact** | | | | | | |
| Worried reaction | Immediate  Soureti 2010 | 413 | Worried response to Heart Age  chisq 7.77, p=0.005 | Worried response to  % risk  chisq 4.05, p=0.044  Diff between group NS |  |  |
| Anxiety about high risk | 6-9 months  van Steenkiste, 2007 | 490 | Anxious at  2 weeks and 6 months  17% 16%Appropriately anxious  2 weeks and 6 months  69% 69% | Anxious at  2 weeks and 6 months  13% 16%  Diff p=NS  Appropriately anxious  2 weeks and 6 months  74% 73%  Diff p=NS |  |  |
| Negative emotional response | 2-4 weeks  Bonner 2015 | 570 | Median (IQR)  Heart Age  3.0 (1.0 – 5.0) | Median (IQR)  Absolute risk %  2.0 (1.0 – 4.7) p=0.40 |  |  |
| Worried scores | 2 weeks  Soureti 2011 | 781 | Planning Tool 4.0  Heart Age 4.7  HA+PTool 4.6 | Control:reported no diff  Overall diff chisq 4.8,  p=0.03 |  |  |
| Decisional conflict | 1 month,  6-9 months  Jaspers 2021 | 303 |  |  | **1m Median (IQR)**  **Abs risk 22(11-30)**  **CVD-free 25 (10-31**)  6m Median (IQR)  Abs risk 22 (9-29)  CVD-free 25 (7-31) | **1m Median (IQR)**  **27 (20-43)**  **p=0.001**  **p=0.02**  6m Median (IQR)  25 (16-38)  P= 0.10 |
| CHD or stroke worry scores | 3 months  Powers 2011 | 89 |  |  | Mean (SD)  CHD worry 39.1 (5.0)  Stroke worry 36.2 (4.9) | Mean (SD)  CHD worry 37.6 (4.9)  p=0.40  Stroke worry 34.6 (4.8)  p=0.69 |
| CHD-related worry  & overall stress | 3 months  Silarova 2019 | 861 | Group 2 vs. control  **Groups 3&4 vs. control**  Overall stress change | Worry Diff  0.12 (-0.13, 0.37)  p= NS  **Worry Diff 0.30 (0.09, 0.52)**  **p= 0.006**  Stress NS for any group |  |  |
| Anxiety score | Average 3 m, 9m, 18m  Nieuwkerk 2012 | 200 |  |  | Mean HADS score (SE)  4.2 (0.2) | Mean HADS score (SE)  5.0 (0.2)  Diff -0.80 (-1.4, -0.2)  P<0.05 |
| Distress score | 12 months  5 years  Christensen 2004 | 390 | GHQ-12 score change  Mean (SD)  12m 0.05 (6.73)  5yr -0.23 (7.19) | GHQ-12 score change  12m -0.16 (6.75)  p=0.603  5 yr -0.39 (7.26)  p=0.727 |  |  |
| **Smoking** | | | | | | |
| Intention to reduce smoking | Immediate,  6 months  Buhse 2015 | 143 | 4/8= 50% | 4/11= 36.4%  Diff 13.6 p=0.552 |  |  |
| Intention to stop | 2 weeks  Bonner 2015 | 469 with follow-up | Absolute Risk median (IQR)= 4.7 (3.7–6.0) | Heart Age median (IQR)= 5.0 (4.0–5.7)  Difference: p=0.67 |  |  |
| Intention to stop | Immediate  Sheridan 2006 | 41 | From 21% to 37%  Absolute difference within group +16%  (-15% to 44%) | Between group difference N/R |  |  |
| Actual achievement of smoking goal | 6 months  Buhse 2015 | 143 | 4/8=50% | 7/12=58%  Diff -8.3%, p=1.0 |  |  |
| Actual smoking change | 2-3 months  Silarova 2019 | 952 | % change  groups 2, 3, 4  -1.26, -0.42, -0.42 | % change 0.84  p>0.02 reported NS |  |  |
| Actual smoking change | 2 months  Svendsen 2018 | 83 | #cigarettes/day (range)  Advice only: 8 (1-12)  Advice + Alert: 5(2-10) | #cigarettes/day (range)  Control: 10 (5-11)  Difference p=0.68 |  |  |
| Actual smoking change | 6 months  van Steenkiste, 2007 | 490 | % smoking 29% | % smoking 23%    Difference NS |  |  |
| Actual smoking change | Mean 3, 9, 18 months  Nieuwkerk, 2012 | 193 | % smoking  30% | % smoking  35%  Difference p>0.05 |  |  |
| Actual smoking cessation | 6-9 months  Nolan 2011 | 93 |  |  | 5/53=9.4% | 3/40=7.5%  Diff NS and N/R |
| Actual smoking change | >12 months  Family Heart 1994  22 | 12,472 | Absolute risk reduction  Overall -4% | Reduction diff (SE)  External control  Men -4.1 (1.8)  Women -3.5 (2.1)  Internal control  Men -4.1 (1.3)  Women -2.9 (1.5) |  |  |
| Actual smoking change | 12 months  Brotons 2021 | 116 | 31.4% | 30.3%  P=0.609 |  |  |
| Actual smoking change | 3 months  Kask-Flight 2021 | 130 | **Mean (SE)**  **-3.8 (1.32)** | **Mean (SE)**  **+2.32 (1.29)**  **Change diff p=0.001** |  |  |
| Actual smoking change | 12 months  Koelewijn-van Loon 2009 | 108 | **Intensive**  **27.3%** | **Minimal**  **17.5%**  **p=0.011** |  |  |
| Actual smoking change | 12 months  Lopez-Gonzalez 2015 | 2844 | **% change**  **Framingham -0.4**  **Heart Age -1.8** | **% change**  **0.9**  **p=0.001** |  |  |
| Actual smoking change | 2, 6, 12+ months  Lovibond 1986 | 18/75 | Reduction %  **Extended 64.4%**  Maximal 29.4 % | Reduction %  **Basic program 29%**  **p=0.001** |  |  |
| Actual smoking change | 12 months  Maron 2008 | 77 | N/R | N/R  incomplete follow-up |  |  |
| Actual smoking change | >12 months  Escortell-Mayor 2020 | 249 |  |  | 19.3% | 21.6%  Adjusted OR  0.866 (-1.537, 0.487) |
| Actual smoking change | Redfern 2020 | 683 |  |  | Non-smoker 91.9% | Non-smoker 88.0%  p= 0.09 |
| Actual smoking change | 2-3 months  Zullig 2014 | 96 |  |  | Current smoking (95%CI) 14% (8-24%) | Current smoking (95%CI)  17% (10-25%) p=0.30 |
| **Physical activity** | | | | | | |
| Intention to improve | Immediate  Sheridan 2006 | 41 | From 29% to 34%  Absolute difference within group +5%  (-12% to 21%) | Difference between groups N/R |  |  |
| Intention to improve | 2 weeks  Bonner 2015 | 570 | Absolute Risk median (IQR)= 4.3 (3.5–5.7) | Heart Age median (IQR)= 4.3 (3.7–5.3)  Difference: p= 0.72  RR 1.12  [95% CI 0.94, 1.33] |  |  |
| Actual change in  P.A./ steps | 2-3 months  Cioe 2021 | 40 | Effect of group @2m  000 (SE 0.25)  Effect of group @3m  -0.10 (SE 0.33) | p=0.98  p=0.76 |  |  |
| Adherence to physical activity program | 6 months  Oddone 2018 | 91 | Program participation rate 40.0% | Program participation rate 23.0%  **OR: 2.3, 95%CI1.5 – 3.6) p<0.001** |  |  |
| Actual change in physical activity | 12 months  Lopez-Gonzalez 2015 | 2844 | **# sessions/week 95%CI)**  **FR: 3.48 (3.35-3.62)**  **HA:3.60 (3.47-3.73)** | **# sessions/week (95%CI)**  **2.23 (2.11-2.36)**  **p<0.001** |  |  |
| **Diet** | | | | | | |
| Intention to reduce saturated fat in 6 months | immediate  Sheridan 2006 | 41 | Within group effect Diff BL to +10% (-8% to 27%) | Between group comparison N/R |  |  |
| Intention to improve diet | 2 weeks  Bonner 2015 | 570 | Absolute risk  12.0% | Heart Age  9.0% Diff p=0.45 |  |  |
| Intention to reduce saturated fat | 5 weeks  Soureti 2011 | 781 | **Planning tool vs. control**  Heart Age vs. control | **OR=21.2,**  **95%CI 2.6, 172.4**  OR=0.04,  95%CI 0.0005, 0.42 |  |  |
| Actual self-reported reduction in saturated fat | 5 weeks  Soureti 2011 | 781 | **Planning Tool OR 11.4, 95% CI 1.86, 69.8** | **Significantly higher than any control groups** |  |  |
| Actual dietary change | 1-3 months  Cioe 2021 | 40 | Both groups improved Rapid Eating and Activity Assessment  Score:  1-m Effect of group:0.1 (SE0.35)  3-m Effect of group: 0.56 (SE0.32) | NS difference between groups  p=0.69  p=0.09 |  |  |
| Actual adherence to diet | 6 months  Nolan 2011 | 448 |  |  | 38.1% | **33.3%**  **Adjusted OR=5.12 (95%CI 3.82, 6.86) p<0.0001** |
| **Medication** | | | | | | |
| **Intention** to start preventive meds if high risk | Immediate  Sheridan 2006 | 41 | Post-intervention absolute diff (95%CI):  Cholesterol  +21% (-5% to 47%)  Antihypertensive  +46% (9% to 75%)  Aspirin  +47% (19% to 72%) | Between group comparisons with control group N/R |  |  |
| Actual change in medication adherence score | 6 to9 months  12-18 months  Tinsel 2013 | 673    607 |  |  | Mean score change  0.07 (SD 9.6)  0.4 (SD 10.7) | Mean score change  -0.3 (SD6.9)  -1.1(SD 6.7)  Mean diff Baseline to 6m  0.670 (95%CI -0.37, 1.72)  P=0.208 |
| Clinician’s Prescribing lipid lowering or BP meds | 6 months  Nolan 2011 | 284 |  |  | Change in prescribing from BL to 6m  42.3% | Change in prescribing from BL to 6m  41.4 Diff p=0.74 |
| Clinician’s Prescribing statins to high-risk patients | 6-9 months  Mortsiefer 2015 | 606 |  |  | Complex: 23.3% (19.7 to 26.9) | Simple: 19.4% (15.8 to 23.0) OR 1.52 (0.89 to 2.62) p= 0.128 |
| Clinician’s initial Prescribing to reduce CVD risk | 12 months  Bucher 2010 | 3,266  Both mostly 1ary | 55/1680=3.3% | 49/1682=2.9%  Reported as NS |  |  |
| **Change in CVD risk factors** | | | | | | |
| Systolic blood pressure | 6-12 months  Lovibond 1986 | 75 | SBP Reduction  6m Maximal -12.9  6m Extended -13.6  12m Maximal -12.1  12m Extended -12.4 | SBP Reduction in  6m basic (control) 8.4  p=0.129  12m basic 7.1  P N/R |  |  |
| Mean systolic blood pressure for high risk >5% | 6-9 months  Mortsiefer 2015 | 606 | Complex: 138.0 (136.4 to 139.7) | Simple: 137.3 (135.6 to 139.1)  Diff -0.7 (-1.7 to 3.1)  p=0.563 |  |  |
| Mean systolic blood pressure by gender | 12 months  FHS 1994 | 5,012 | **Men 131.6**  **Women 123.2** | **External ctrl Diff (SE)**  **Men 138.8 -7.5(1.2)**  **Women 130.8 -7.7(1.4)**  **internal ctrl Diff (SE)**  **Men 139.0 -7.3(0.8)**  **Women 129.6 -6.2(0.9)** |  |  |
| Mean systolic Blood pressure | 12 months  Maron 2008 | 77 |  |  | Mean (SD)  133 (3.0) | Mean (SD)  128 (3.0)  P NS |
| Mean systolic Blood pressure | 3 months  Mitchell 2005 | 52 practices |  |  | 149.8 | Control 1 148.0  Control 2 154.3 p NS |
| Lipids for patients initially classed as hyper | 6 months  Lovibond 1986 | 75 | Mean cholesterol reduction 0.5 mmol/L  Mean triglycerides reduction 0.9 mmol/L | No differences found between groups (no data reported for controls) |  |  |
| Change in HDL cholesterol | 12 months  Maron 2008 | 133 | HDL reduction -0.9 | HDL reduction -0.1  p=0.026 |  |  |
| % achieving LDL goal | 9 months  Persell 2013 | 435 | % with LDL <30 mg/dl lower than baseline 11.0 | % with LDL lower than baseline 11.0  p=0.96 |  |  |

1. Type of comparator of interest for this systematic review [↑](#footnote-ref-2)
2. Type of intervention of interest for this systematic review [↑](#footnote-ref-3)
3. Usual care with an additional component (e.g., education, training) [↑](#footnote-ref-4)
4. With risk information provided in another format/method [↑](#footnote-ref-5)
5. Adopted from the Behaviour-Change Wheel model [↑](#footnote-ref-6)
6. Face-to-face [↑](#footnote-ref-7)
7. Including online/phone/mail [↑](#footnote-ref-8)
8. Printed Informational materials given to educate/inform patients (e.g., leaflet, summary report) [↑](#footnote-ref-9)
9. Paper-based tools used by clinicians either with the patient or alone (e.g., decision aids, risk summary report) [↑](#footnote-ref-10)
10. Website, online tool, automated [↑](#footnote-ref-11)
11. Communicating the risk verbally without any tool or material

    ^P^ Patient level

    ^C^ Clinician level [↑](#footnote-ref-12)
